# *Mycobacterium abscessus* multispacer sequence typing

**DOI:** 10.1186/1471-2180-13-3

**Published:** 2013-01-07

**Authors:** Mohamed Sassi, Imen Ben Kahla, Michel Drancourt

**Affiliations:** 1 Unité de Recherche sur les Maladies Infectieuses et Tropicales Emergentes (URMITE), UMR CNRS 7278, IRD 198, INSERM 1095. Faculté de médecine, 27, Boulevard Jean Moulin-Cedex 5, Marseille, France; 2 Laboratoire de Microbiologie et d’Immunologie, UR02/SP13, CHU Farhat Hached Sousse, Tunisie, France

**Keywords:** *Mycobacterium*, *Mycobacterium abscessus*, *Mycobacterium massiliense*, *Mycobacterium bolletii*, Multispacer sequence typing, Genotyping

## Abstract

**Background:**

*Mycobacterium abscessus* group includes antibiotic-resistant, opportunistic mycobacteria that are responsible for sporadic cases and outbreaks of cutaneous, pulmonary and disseminated infections. However, because of their close genetic relationships, accurate discrimination between the various strains of these mycobacteria remains difficult. In this report, we describe the development of a multispacer sequence typing (MST) analysis for the simultaneous identification and typing of *M. abscessus* mycobacteria. We also compared MST with the reference multilocus sequence analysis (MLSA) typing method.

**Results:**

Based on the *M. abscessus* CIP104536^T^ genome, eight intergenic spacers were selected, PCR amplified and sequenced in 21 *M. abscessus* isolates and analysed in 48 available *M. abscessus* genomes. MST and MLSA grouped 37 *M. abscessus* organisms into 12 and nine types, respectively; four formerly “*M. bolletii*” organisms and *M. abscessus* M139 into three and four types, respectively; and 27 formerly “*M. massiliense*” organisms grouped into nine and five types, respectively. The Hunter-Gaston index was off 0.912 for MST and of 0.903 for MLSA. The MST-derived tree was similar to that based on MLSA and *rpoB* gene sequencing and yielded three main clusters comprising each the type strain of the respective *M. abscessus* sub-species. Two isolates exhibited discordant MLSA- and *rpoB* gene sequence-derived position, one isolate exhibited discordant MST- and *rpoB* gene sequence-derived position and one isolate exhibited discordant MST- and MLSA-derived position. MST spacer n°2 sequencing alone allowed for the accurate identification of the different isolates at the sub-species level.

**Conclusions:**

MST is a new sequencing-based approach for both identifying and genotyping *M. abscessus* mycobacteria that clearly differentiates formerly “*M. massiliense*” organisms from other *M. abscessus* subsp. *bolletii* organisms.

## Background

*Mycobacterium abscessus* mycobacteria are increasingly being cultured from respiratory tract specimens collected from patients with chronic pulmonary diseases, including cystic fibrosis [1-9]. These mycobacteria are also responsible for skin and soft-tissue infections following surgical and cosmetic practices [10-12] and catheter-related bacteremia [13,14]. These infections are particularly critical for immune-compromised patients and may be fatal [15]. Water is suspected as a source of infection, as *M. abscessus* mycobacteria have been isolated from tap water [16]. Moreover, *M. abscessus* mycobacteria have been shown to be resistant to water-borne free-living amoebae [17,18]. *M. abscessus* infections are also associated with treatment failure owing, due to the natural broad-spectrum resistance to antibiotics in addition to acquired resistance, with subtle differences in the antibiotic susceptibility pattern being observed among isolates [19].

Indeed, *M. abscessus* is comprised of a heterogeneous group of mycobacteria currently classified into *M. abscessus* subsp. *abscessus* and *M. abscessus* subsp. *bolletii*[20,21], with the later subspecies accommodating mycobacteria previously identified as “*Mycobacterium bolletii*” or “*Mycobacterium massiliense*” [18,22]*.* However, these organisms are nearly indistinguishable using phenotypic tests including the mycolic acid pattern analysis and share 100% 16S rRNA gene sequence similarity [20]. They were initially differentiated on the basis of >3% *rpoB* gene sequence divergence and different antimicrobial susceptibility patterns [23,24]. Nevertheless, confusing results based on *rpo*B sequencing have been reported [21], and combining sequencing of the *rpo*B, *hsp65* and *secA* genes has been advocated for the optimal identification of the *M. abscessus* mycobacteria [25].

To further decrypt the diversity and genetic relationships among *M. abscessus* organisms, we investigated a collection of reference, sequenced genomes and clinical *M. abscessus* isolates using multispacer sequence typing (MST), which is a sequencing-based approach previously used for the species identification and genotyping of Mycobacteria, including *Mycobacterium avium*[26] and *Mycobacterium tuberculosis*[27] and non-mycobacterial pathogens, such as *Yersinia pestis*[28], *Rickettsia prowazekii*[29] and *Bartonella quintana*[30]*.* This approach was here compared with multilocus sequence analaysis which relies the sequencing of 5–8 genes (21, 25), and *rpoB* genes sequencing (23, 24).

## Methods

### Bacterial isolates

Reference *M. abscessus* CIP104536^T^, *M. abscessus* DSMZ44567 (German Collection of Microorganisms and Cell Cultures, Braunschweig, Germany), *M. abscessus* subsp. *bolletii* CIP108541^T^ (herein referred as “*M. bolletii*”) and *M. abscessus* subsp. *bolletii* CIP108297^T^ (herein referred as “*M. massiliense*” [23]) were used in this study. In addition, a collection of 17 *M. abscessus* clinical isolates from the mycobacteria reference laboratory of the Méditerranée Infection Institute, Marseille, France were also studied (Table 1). All of the mycobacteria were grown in 7H9 broth (Difco, Bordeaux, France) enriched with 10% OADC (oleic acid, bovine serum albumin, dextrose and catalase) at 37°C. As for the identification, DNA extraction and *rpo*B partial sequence-based identification were performed using the primers MYCOF and MYCOR2 (Table 1) as previously described [24]. In addition, the *rpoB* gene sequence retrieved from 48 *M. abscessus* sequenced genomes was also analysed (Additional file 1) ( http://www.ncbi.nlm.nih.gov/).

**Table 1:** Spacers characteristics used in this study

**Name**	Genome position*	Framing genes*	**PCR primers**	**PCR product size (bp)**
Spacer 1	106145-106396	MAB_0104:enoyl-CoA hydratase/isomerise	F : GGGATGCGCAGATGACGGGG	506
		MAB_0105c:oxidoreductase	R : GCTACCCCGAATGGGGCACG	
Spacer 2	173727-173985	MAB_0176:antigen 85-A precursor	F : TCGAGTTTCCTCCGGGCGGT	438
		MAB_0177:antigen 85-A/B/C precursor	R: AATCCAGGCAGAACGGCCGC	
Spacer 3	422777-423027	MAB_0423c:hypothetical protein	F: GCCATTGCTGTCCGTGCGGT	344
		MAB_0424:putative protease	R : GCCGCGAACAGGCCAAACAG	
Spacer 4	494411-494670	MAB_0495c:hypothetical protein	F: CGCCCTTGCGCAGGAGTGAT	528
		MAB_0496c:hypothetical protein	R: GCCTGGTTCGGACGGTGACG	
Spacer 5	761805-762060	MAB_0761c:putative 3-hydroxyacyl-CoA dehydrogenase	F : ACCACATCGGCGAGCGTGTG	545
		MAB_0762:hypothetical protein	R : CCAACACCGGGTCGCGGTAC	
Spacer 6	771170-771436	MAB_0772c:hypothetical protein	F : CGTCGGTCTTGCCGACCGTC	600
		MAB_0773:hypothetical protein	R : GGCGCCGACGATCTAGCACC	
Spacer 7	880381-880639	MAB_0887c:hypothetical protein	F: CGGCAGTGCAAGGTGCGTTG	519
		MAB_0888c:putative fumarylacetoacetase	R : GCACCGTGTCCGGTCCTCAG	
Spacer 8	959422-959678	MAB_0950c:putative amino acid permease family protein	F: GGGGCGTATGCGCCGTTACC	474
		MAB_0951:putative aminoglycoside phosphotransferase	R : CGAACGCGCTGTGATTCGGC	
Spacer 9	1002935-1003200	MAB_0995:hypothetical protein	F : GGCCGCGACAAGCTGATCGT	684
		MAB_0997c:hypothetical protein	R: ATGCAGGGCACCGTGCGTAG	
Spacer 10	1216613-1216879	MAB_1201c:transcription elongation factor GreA	F: CGTTCTCGCGCAGGTCTCCC	517
		MAB_1202c:hypothetical protein	R: CCGAACGATCCGTGCCGGTC	
Spacer 11	1818877-1819188	MAB_1818:hypothetical protein	F: AGCCAACTGCCATGGCGCTT	495
		MAB_1819c:hypothetical protein	R : ACCGAGACGTCATGCACCGC	

* With reference to *M. abscessus* ATCC 19977 genome.

### Reference MLSA typing

Fragments from five housekeeping genes *argH* (argininosuccinate lyase), *cya* (adenylate cyclase), *murC* (UDP N-acetylmuramate-L-Ala ligase, *pta* (phosphate acetyltransferase) and *purH* (phoshoribosylminoimiazolcarboxylase ATPase subunit) were amplified using the sets of primers as previously described (21). The sequences of each one of these five housekeeping genes retrieved from 48 *M. abscessus* sequenced genomes, were also included in the MLSA analysis (Additional file 1).

### MST analysis

Sequences of the whole intergenic spacers were extracted from the reference *M. abscessus* CIP104536^T^ (ATCC19977) genome (GenBank accession CU458896.1) using the perl script software and a total of 8 spacers with a 200-700-bp sequence size were further used in analysis. For each of these 8 spacers, specific PCR primers were designed using Primer3 software v 0.4.0 ( http://frodo.wi.mit.edu/primer3) and tested *in silico* for specificity using BLAST software ( http://www.ncbi.nlm.nih.gov). The PCR conditions were first optimized using DNA extracted from the reference *M. abscessus*, “*M. bolletii”* and *“M. massiliense”* isolates before analysis of DNA extracted from the 17 clinical isolates (Table 1). The PCR amplifications were performed in a 50 μl PCR mixture containing 5 μl 10x buffer (Qiagen, Courtaboeuf, France), 200 mM each dNTP, 1.5 mM MgCl_2_, 1.25 U HotStarTaq polymerase (Qiagen), 1 μl each primer (10 pM), 33 μl nuclease-free water and 5 μl DNA template. The amplification program consisted of an initial 15 min denaturation step at 95°C followed by 40 cycles of 30 s at 95°C, 30 s at 60°C and 1 min at 72°C; the amplification was completed by a final 5-min elongation step at 72°C. Negative controls consisting of PCR mixture without DNA template were included in each PCR run. The products were visualized by gel electrophoresis, purified using a MultiScreen PCR filter plate (Millipore, Molsheim, France) and sequenced in both directions using the BigDye Terminator sequencing kit (Applied Biosystems, Villebon-sur-Yvette, France), as previously described [27]. The sequences were edited using the ChromasPro software (version 1.42; Technelysium Pty Ltd), aligned using Clustal W (MEGA 5 software) and compared with the reference *M. abscessus* ATCC 19977 sequences (GenBank accession CU458896.1). For MST and MLSA discrimination power was calculated using the Hunter-Gaston Index [31]:

(1) $$\text{DI}=1-\left[\frac{1}{N\left(N-1\right)}\sum_{j=1}^{8}n_{j}\left(n_{j}-1\right)\right]$$

where *D* is the numerical index of discrimination, *N* is the total number of isolates in the sample population, *s* is the total number of different types and *nj* is the number of isolates belonging to the *j*th type.

### Phylogenetic analysis

Phylogenetic trees were constructed based on *rpoB* gene, concatenated MLSA genes, concatenated spacers and MST spacer n°2 sequences using the neighbor–joining method with Kimura’s two-parameter (K2P) distance correction model with 1000 bootstrap replications in the MEGA version 5 software package [32]. The *rpo*B gene sequence-based tree was rooted using *M. chelonae* strain CIP 104535^T^ and *M. immunogenum* strain CIP 106684^T^*rpo*B gene sequences. A heatmap was constructed using the R statistical software based on the spacer profile as a distance matrix.

## Results and discussion

### rpoB identification and rpoB tree

**Figure 1:**
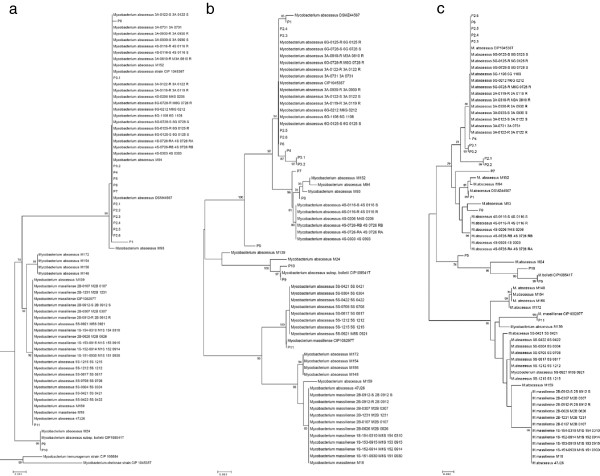
**Phylogenetic tree based on *rpoB *gene sequence (a); based on the concatenated five MLSA gene sequences (b); and based on the concatenated eight polymorphic spacers (c).**

The identification of *M. abscessus* CIP104536^T^, *M. abscessus* DSMZ44567, *M. bolletii* CIP108541^T^ and *M. massiliense* CIP108297^T^ was confirmed by partial *rpo*B sequencing. The sequences were deposited in the GenBank database (GenBank accession: KC352778 - KC352795). Isolates P1, P2.1, P2.2, P2.3, P2.4, P2.5, P3.1, P3.2, P4, P5, P6, P7 and P8 exhibited 99% *rpo*B sequence similarity with *M. abscessus* ATCC19977^T^ and were identified as *M. abscessus*. Isolates P9 and P10 exhibited 99% *rpo*B sequence similarity with “*M. bolletii”* CIP108541^T^ and were identified as “*M. bolletii”* whereas isolate P11 exhibited 99% *rpo*B sequence similarity with “*M. massiliense”* CIP108297^T^ and was identified as “*M. massiliense*”*.* A total of 23 *M. abscessus* sequenced genomes were identified as *M. abscessus* since they exhibited 98 to 100% similarity with the *M. abscessus* type strain *rpoB* partial gene sequence. *M. abscessus* M24 shared 99% similarity with the *M. bolletii* type strain partial *rpoB* gene sequence. A total of 26 *M. abscessus* and “*M. massiliense”* sequenced genomes shared 99% to 100% similarity with “*M. massiliense”* partial *rpoB* gene sequence. The tree built from 69 partial *rpoB* gene sequences showed three distinct groups, each comprising the type strain (Figure 1a).

### Reference MLSA analysis

Fragments for the expected size were amplified and sequenced for the five MLSA genes. The sequences were deposited in the GenBank database (GenBank accession: KC352742 - KC352759, KC352760 - KC352777, KC352796 - KC352813, KC352814 - KC352831, KC352832 - KC352849). Concatenation of the five sequences yielded a total of 19 different types, including 9 types for 37 *M. abscessus* organisms, four types for 4 *“M. bolletii”* organisms and *M. abscessus* M139 and five types for 27 *“M. massiliense”* organisms. The Hunter-Gaston Index for MLSA was of 0.903. The MLSA tree based on the five gene concatened sequences showed three principal clusters, i.e. a *M. abscessus* cluster, a “*M. bolletii”* cluster and a “*M. massiliense”* cluster (Figure 1b). Latter cluster comprised of five sub-clusters with “*M. massiliense”* type strain and P11 strain sub-clustering together close to *M. abscessus* 5S strain. Also, MLSA-derived tree clustered *M. abscessus* M139 strain and P5 strain respectively identified as “*M. massiliense”*, close to the “*M. bolletii”* whereas both strains clustered with *M. abscessus* in the *rpoB* gene sequence-derived tree.

### MST analysis

Analysis of the reference *M. abscessus* ATCC 19977 complete genome sequence yielded 3538 intergenic spacers with > 300 spacers were 200–700 bp in length. Successful PCR sequencing was achieved for 8 spacers in all the isolates studied; the sequences were deposited in the GenBank database (GenBank accession: KC352850 - KC352890). In *M. abscessus* isolates, including the 37 sequenced genomes, the spacer sequence variability was generated by one to 12 single nucleotide polymorphisms (SNPs) (spacers n°1 and n°8), one to 18 SNPs and one to two nucleotide deletions (spacer n°2), one to two SNPs (spacers n°3 and n°7) and nucleotide insertion (spacers n°2 and n°5). In “*M. bolletii*” isolates, the spacer sequence polymorphisms were generated by one SNP for spacer n°1, two SNPs and one deletion for spacer n°2, two SNPs for spacer n°3 and nine SNPs for spacer n°7. In “*M. massiliense*” isolates, including 28 sequenced genomes, the spacer sequence polymorphism were generated by nine SNPs and one insertion (spacer n°1), one insertion (spacer n°3), five SNPs and two insertions (spacer n°4), one SNP (spacer n°5) and two SNPs (spacer n°7). Concatenation of the eight spacer sequences yielded a total of 24 types, with the 37 *M. abscessus* organisms grouped into 12 spacer types, four formerly “*M. bolletii*” organisms grouped into three spacer types and 28 formerly “*M. massiliense*” organisms grouped into nine spacer types. This yielded a Hunger-Gaston Index of 0.912. Spacer n°5 was found to be the most variable of the eight spacers under study, exhibiting 13 different alleles (Table 2). When combining the eight spacer sequences, a unique MST profile for each reference isolate was obtained, i.e., MST1 and MST2 for *M. abscessus* CIP104536^T^ and *M. abscessus* DSMZ44567 respectively, MST13 for “*M. bolletii”* CIP108541^T^ and MST16 for “*M. massiliense”* CIP108297^T^. At the sequence level, we found that MST1 and MST2 genotypes differ by at most nine SNPs, whereas MST1 differed from MST13 by up to 18 SNPs, one insertion and two deletions and from MST16 by 14 SNPs, 11 deletions and two insertions (supplementary material). The 17 clinical *M. abscessus* isolates were grouped into eight MST types, named MST1 to MST8, with five *M. abscessus* isolates exhibiting the *M. abscessus* CIP104536^T^ MST1 genotype and one isolate (P1 strain) exhibiting the *M. abscessus* DSMZ44567 MST2 genotype. The P9 “*M. bolletii*” clinical isolate yielded the MST13 genotype in common with the reference “*M. bolletii*” CIP108541^T^, whereas the P10 “*M. bolletii*” clinical isolate yielded a unique MST14 genotype that differ from MST13 by two SNPs in spacer n°1. *M. abscessus* M24 yielded the MST15 and differed from MST13 by four polymorphic spacers. In “*M. massiliense”* nine different profiles were generated MST 16 to MST24. The P11 “*M. massiliense*” clinical isolate shared the MST16 genotype with the reference “*M. massiliense*” CIP108297^T^. “*M. massiliense”* 2B isolate, “*M. massiliense”* 1S isolate and “*M. massiliense”* M18 isolate shared the same MST profile (MST17). *M. abscessus* 5S isolate exhibited the MST21 profile.

**Table 2:** **Spacers allelic polymorphism and MST **^a ^**genotypes of *M. abscessus*, “*M. bolletii” *and “*M. massiliense” *isolates**

**Isolates**	**Spacer1**	**Spacer2**	**Spacer3**	**Spacer4**	**Spacer5**	**Spacer6**	**Spacer7**	**Spacer8**	**Genotype**
***M.abscessus_*ATCC19977_CIP104536T**	1	1	1	1	1	1	1	1	**1**
***M.abscessus_*DSMZ44567**	2	1	2	2	2	1	2	1	**2**
*P1*	2	1	2	2	2	1	2	1	**2**
*P2.1*	1	2	1	3	1	1	2	2	**3**
*P2.2*	1	2	1	3	1	1	2	2	**3**
*P2.3*	1	1	1	1	1	1	1	1	**1**
*P2.4*	1	1	1	1	1	1	1	1	**1**
*P2.5*	1	1	1	1	1	1	1	1	**1**
*P2.6*	1	1	1	1	1	1	1	1	**1**
*P3.1*	3	1	2	1	1	1	2	1	**4**
*P3.2*	3	1	2	1	1	1	2	1	**4**
*P4*	1	1	1	1	1	1	1	2	**5**
*P5*	1	1	1	1	3	1	2	1	**6**
*P6*	1	1	1	1	1	1	1	1	**1**
*P7*	4	1	2	4	4	1	2	1	**7**
*P8*	4	1	2	4	4	1	3	1	**8**
***M.abscessus_3A-0930-R_3A_0930_R***	1	1	1	1	1	1	1	1	**1**
***M.abscessus_3A-0930-S_3A_0930_S***	1	1	1	1	1	1	1	1	**1**
***M.abscessus_3A-0122-S_3A_0122_S***	1	1	1	1	1	1	1	1	**1**
***M.abscessus_3A-0731_3A_0731***	1	1	1	1	1	1	1	1	**1**
***M.abscessus_3A-0122-R_3A_0122_R***	1	1	1	1	1	1	1	1	**1**
***M.abscessus_3A-0119-R_3A_0119_R***	1	1	1	1	1	1	1	1	**1**
***M.abscessus_6G-0728-R_M6G_0728_R***	1	1	1	1	1	1	1	1	**1**
***M.abscessus_6G-0212_M6G_0212***	1	1	1	1	1	1	1	1	**1**
***M.abscessus _6G-1108_6G_1108***	1	1	1	1	1	1	1	1	**1**
***M.abscessus _6G-0728-S_6G_0728_S***	1	1	1	1	1	1	1	1	**1**
***M.abscessus_6G-0125-R_6G_0125_R***	1	1	1	1	1	1	1	1	**1**
***M.abscessus _6G-0125-S_6G_0125_S***	1	1	1	1	1	1	1	1	**1**
***M.abscessus_4S-0116-S_4S_0116_S***	5	1	2	5	5	2	2	2	**9**
***M.abscessus_4S-0116-R_4S_0116_R***	5	1	2	5	5	2	2	2	**9**
***M.abscessus_4S-0206_M4S_0206***	5	1	2	5	5	2	2	2	**9**
***M.abscessus_4S-0726-RB_4S_0726_RB***	5	1	2	5	5	2	2	2	**9**
***M.abscessus_4S-0303_4S_0303***	5	1	2	5	5	2	2	2	**9**
***M.abscessus_4S-0726-RA_4S_0726_RA***	5	1	2	5	5	2	2	2	**9**
***M.abscessus_M93***	3	1	2	6	6	1	2	3	**10**
***M.abscessus_M94***	2	1	2	2	7	1	4	2	**11**
***M.abscessus_M152***	2	1	2	7	7	1	2	3	**12**
***M.bolletti_*CIP108541T**	6	3	3	3	8	1	5	2	**13**
*P9*	6	3	3	3	8	1	5	2	**13**
*P10*	7	4	1	3	8	1	2	2	**14**
***M.abscessus_M24***	8	3	4	8	8	1	2	2	**15**
***M.massilliense_*CIP108297T**	5	5	5	9	9	1	6	3	**16**
*P11*	5	5	5	9	9	1	6	3	**16**
***M.massiliense _2B-0912-S_2B_0912_S***	9	5	6	10	10	2	7	3	**17**
***M.massiliense_2B-030_ M2B_0307***	9	5	6	10	10	2	7	3	**17**
***M.massiliense_2B-0912-R_2B_0912_R***	9	5	6	10	10	2	7	3	**17**
***M.massiliense_2B-0626_M2B_0626***	9	5	6	10	10	2	7	3	**17**
***M.massiliense_2B-1231_M2B_1231***	9	5	6	10	10	2	7	3	**17**
***M.massiliense_2B-0107_M2B_0107***	9	5	6	10	10	2	7	3	**17**
***M.massiliense _1S-154-0310_M1S_154_0310***	9	5	6	10	10	2	7	3	**17**
***M.massiliense_1S-152-0914_M1S_152_0914***	9	5	6	10	10	2	7	3	**17**
***M.massiliense_1S-153-0915_M1S_153_0915***	9	5	6	10	10	2	7	3	**17**
***M.massiliense_1S-151-0930_M1S_151_0930***	9	5	6	10	10	2	7	3	**17**
***M.massiliense _M18***	9	5	6	10	10	2	7	3	**17**
***M.abscessus_M159***	9	6	6	9	10	3	7	4	**18**
***M.abscessus_47J26***	9	5	6	6	11	4	7	3	**19**
***M.abscessus_M172***	10	7	2	9	12	3	8	5	**20**
***M.abscessus_M154***	10	7	2	9	12	3	8	5	**20**
***M.abscessus_5S-1215_5S_1215***	11	5	2	6	13	2	6	2	**21**
***M.abscessus_5S-1212_5S_1212***	11	5	2	6	13	2	6	2	**21**
***M.abscessus_5S-0817_5S_0817***	11	5	2	6	13	2	6	2	**21**
***M.abscessus_5S-0708_5S_0708***	11	5	2	6	13	2	6	2	**21**
***M.abscessus_5S-0422_5S_0422***	11	5	2	6	13	2	6	2	**21**
***M.abscessus_5S-0304_5S_0304***	11	5	2	6	13	2	6	2	**21**
***M.abscessus_5S-0421_5S_0421***	11	5	2	6	13	2	6	2	**21**
***M.abscessus_M156***	10	7	2	11	12	3	9	5	**22**
***M.abscessus_M148***	10	7	2	11	12	3	9	5	**23**
***M.abscessus_M139***	10	5	2	11	14	3	10	3	**24**
DI	**0.8295**	**0.6228**	**0.6969**	**0.8001**	**0.8371**	**0.6038**	**0.8084**	**0.7158**	**0.912**

^a^*MST* = Multispacer Sequence Typing. ^b^ isolates were listed with reference to their corresponding patient, for example *P1* = isolate 1 from patient 1, *P2.1* = isolate 1 from patient 2, etc. ^c^*DI* = Discrimination index.

### MST based tree and comparaison with rpoB identification and MLSA analysis

**Figure 2:**
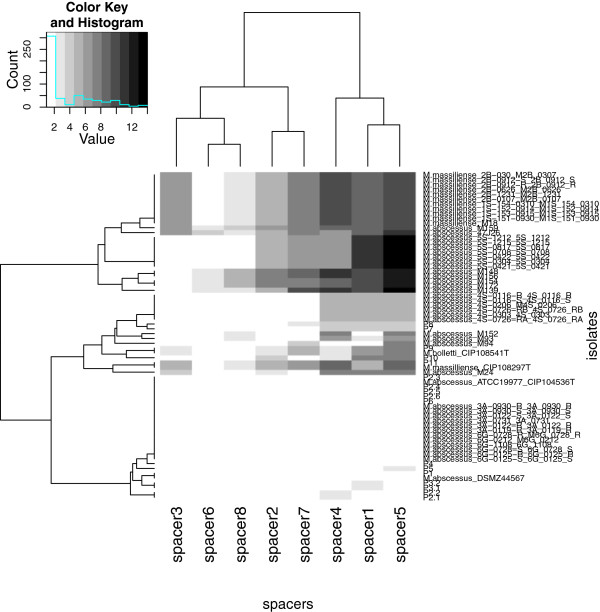
**Heatmap and clustering of *M. abscessus *mycobacteria under study based in difference of profile.**

**Figure 3:**
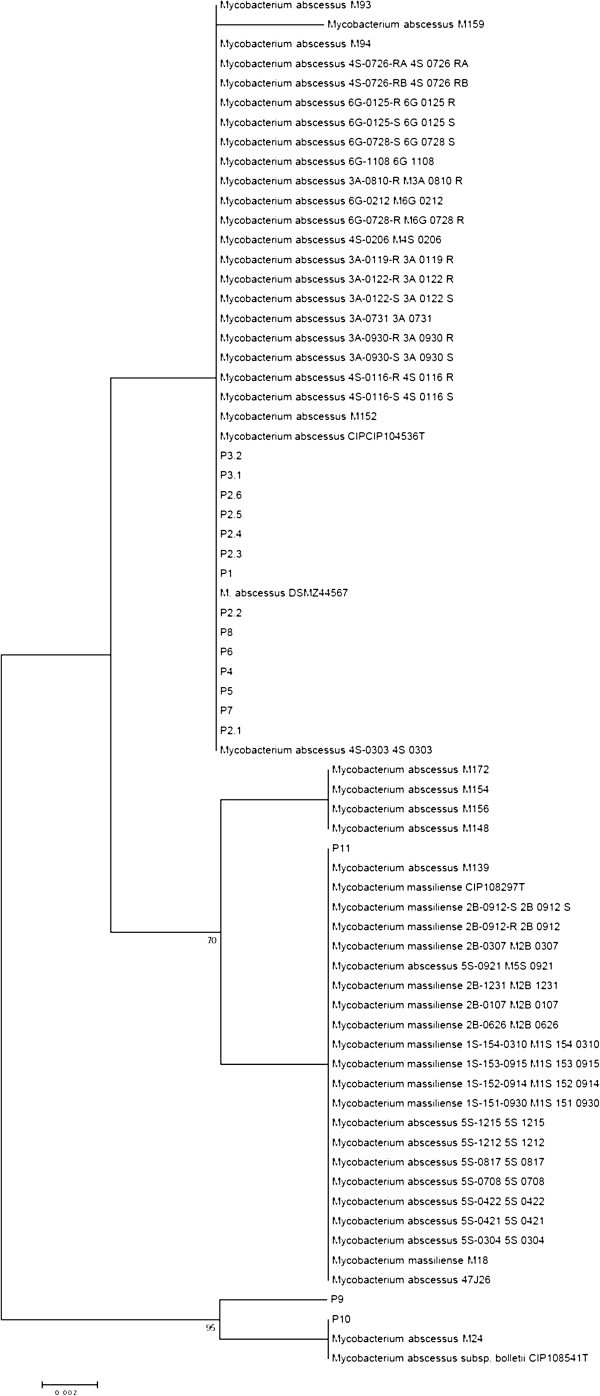
Phylogenetic tree based on MST spacer n°2 sequence.

The MST-phylogenetic tree clustered isolates from patients P1 to P8 with *M. abscessus* reference strain, isolates from P9 and P10 with “*M. bolletii*” and isolate from P11 with “*M. massiliense*”, in agreement with their *rpo*B sequence-based identification and MLSA analysis (Figure 1c). The MST, MLSA and *rpo*B phylogenetic trees separated the *M. abscessus* isolates into three principal clusters depicted by *M. abscessus,* “*M. bolletii*” and “*M. massiliense*” isolates (Figure 1a, b and c). However, MST resolved “*M. bolletii”* cluster into two sub-clusters formed by isolate P5 and all of the other *M. bolletii* isolates with a 76% bootstrap value, wich is discordant with MLSA and *rpoB* based tree. Each cluster or sub-cluster of the *M. abscessus* isolates corresponded to different genotypes. The “*M. massiliense*” cluster was more disperse and divided into six sub-clusters with isolate P11 and “*M. massiliense*” type strain sub-clustering alone. The results of this analysis were consistent for 67 isolates and inconsistent for two isolates P5 and *M. abscessus* M139. A heatmap incorporating all spacer patterns into a matrix further demonstrated that spacer n°2 was the most discriminating spacer (Figure 2). Hence, the tree based on the spacer n°2 sequence also discriminated the three *M. abscessus*, “*M. bolletii*” and “*M. massiliense*” clusters (Figure 3). This discrimination potential makes spacer n°2 a useful new tool for the accurate identification of *M. abscessus* subspecies. Furthermore, these data indicated that it was readily possible to discriminate isolates that would have been identified as “*M. bolletii*” [26] or “*M. massiliense*” [23] using a previous taxonomy proposal and are now grouped as *M. abscessus* subsp. *bolletii* according to a recent taxonomy proposal [20,21].

## Conclusion

We herein developed a sequencing-based MST genotyping technique that allows the accurate identification and discrimination of *M. abscessus* mycobacteria. Therefore, MST could be added to the panel of molecular methods currently available for genotyping *M. abscessus* mycobacteria, with the advantages that MST is a PCR and sequencing-based technique, thereby providing a robust and accurate result without requiring a high DNA concentration and purity, as is the case for pulsed-field gel electrophoresis (PFGE) [5] and randomly amplified polymorphic DNA (RAPD) [33]. Furthermore, MST targets intergenic spacers, which undergo less evolutionary pressure and are thus more variable than the housekeeping genes targeted in multilocus sequence typing [21]. Also, MST incorporating sequencing is an open approach to described new genotypes more versatile than counting the number of tandem repeats [34]. We propose that MST could be incorporated into a polyphasic molecular approach to resolve the phylogenetic relationships of difficult-to-identify *M. abscessus* isolates [35]. Combining MST data with phylogenetic analyses clearly indicated that *M. abscessus* heterogeneity spans beyond the current two *M. abscessus* subspecies, as two “*M. massiliense*” isolates were readily discriminated from the other “*M. bolletii”* isolates [21]. These data, therefore, question the current nomenclature of *M. abscessus* mycobacteria, which incorporates mycobacteria previously recognized as “*M. bolletii*” and “*M. massiliense*” as “*M. abscessus* subsp. *bolletii*”. The data presented here indicate that this nomenclature masks the underlying diversity of *M. abscessus* mycobacteria, potentially hampering the recognition of microbiological, epidemiological and clinical particularities that are linked to each subspecies. The elevation of “*M. massiliense*” as a new *M. abscessus* subspecies would accommodate the data produced in the present study [24].

## Competing interests

The authors declare that they have no competing interest.

## Authors’ contributions

MS and IBK performed molecular analyses. MD designed the study. IBK, MS and MD interpreted data and wrote the draft. All authors read and approved the final manuscript.

## Supplementary Material

Additional file 1 ***rpoB *and MLSA genes accession Number of 49 sequenced genomes.** Click here for file

## References

[B1] Griffith DE Girard WM Wallace RJ Jr Clinical features of pulmonary disease caused by rapidly growing mycobacteria. An analysis of 154 patients Am Rev Respir Dis 1993 147 1271 1278 8484642 10.1164/ajrccm/147.5.1271

[B2] Pierre-Audigier C Ferroni A Sermet-Gaudelus I Le Bourgeois M Offredo C Vu-Thien H Fauroux B Mariani P Munck A Bingen E Guillemot D Quesne G Vincent V Berche P Gaillard JL Age-related prevalence and distribution of nontuberculous mycobacterial species among patients with cystic fibrosis J Clin Microbiol 2005 43 3467 3470 10.1128/JCM.43.7.3467-3470.2005 16000480 PMC1169165

[B3] Olivier KN Weber DJ Wallace RJ Jr Faiz AR Lee JH Zhang Y Brown-Elliot BA Handler A Wilson RW Schechter MS Edwards LJ Chakraborti S Knowles MR Nontuberculous mycobacteria. I: multicenter prevalence study in cystic fibrosis Am J Respir Crit Care Med 2003 167 828 834 10.1164/rccm.200207-678OC 12433668

[B4] Chalermskulrat W Sood N Neuringer IP Hecker TM Chang L Rivera MP Paradowski LJ Aris RM Non-tuberculous mycobacteria in end stage cystic fibrosis: implications for lung transplantation Thorax 2006 61 507 513 10.1136/thx.2005.049247 16601086 PMC2111233

[B5] Jönsson BE Gilljam M Lindblad A Ridell M Wold AE Welinder-Olsson C Molecular epidemiology of Mycobacterium abscessus, with focus on cystic fibrosis J Clin Microbiol 2007 45 1497 1504 10.1128/JCM.02592-06 17376883 PMC1865885

[B6] Levy I Grisaru-Soen G Lerner-Geva L Kerem E Blau H Bentur L Aviram M Rivlin J Picard E Lavy A Yahav Y Rahav G Multicenter cross-sectional study of nontuberculous mycobacterial infections among cystic fibrosis patients Israel Emerg Infect Dis 2008 14 378 384 10.3201/eid1403.061405 PMC2570835 18325250

[B7] Griffith DE Emergence of nontuberculous mycobacteria as pathogens in cystic fibrosis Am J Respir Crit Care Med 2003 167 810 812 10.1164/rccm.2301001 12623856

[B8] Roux AL Catherinot E Ripoll F Soismier N Macheras E Ravilly S Bellis G Vibet MA Le Roux E Lemonnier L Gutierrez C Vincent V Fauroux B Rottman M Guillemot D Gaillard JL Jean-Louis Herrmann for the OMA Group Multicenter study of prevalence of nontuberculous mycobacteria in patients with cystic fibrosis in france J Clin Microbiol 2009 47 4124 4128 10.1128/JCM.01257-09 19846643 PMC2786646

[B9] Uyan ZS Ersu R Oktem S Cakir E Koksalan OK Karadag B Karakoc F Dagli E Mycobacterium abscessus infection in a cystic fibrosis patient: a difficult to treat infection Int J Tuberc Lung Dis 2010 14 250 251 20074421

[B10] Furuya EY Paez A Srinivasan A Cooksey R Augenbraun M Baron M Brudney K Della-Latta P Estivariz C Fischer S Flood M Kellner P Roman C Yakrus M Weiss D Granowitz EV Outbreak of mycobacterium abscessus wound infections among “lipotourists” from the United States who underwent abdominoplasty in the Dominican Republic Clin Infect Dis 2008 46 1181 1188 10.1086/529191 18444853

[B11] Koh SJ Song T Kang YA Choi JW Chang KJ Chu CS Jeong JG Lee JY Song MK Sung HY Kang YH Yim JJ An outbreak of skin and soft tissue infection caused by Mycobacterium abscessus following acupuncture Clin Microbiol Infect 2010 16 895 901 19694761 10.1111/j.1469-0691.2009.03026.x

[B12] Viana-Niero C Lima KV Lopes ML Rabello MC Marsola LR Brilhante VC Durham AM Leão SC Molecular characterization of Mycobacterium massiliense and Mycobacterium bolletii in isolates collected from outbreaks of infections after laparoscopic surgeries and cosmetic procedures J Clin Microbiol 2008 46 850 855 10.1128/JCM.02052-07 18174307 PMC2268380

[B13] Petrini B Mycobacterium abscessus: an emerging rapid-growing potential pathogen APMIS 2006 114 319 328 10.1111/j.1600-0463.2006.apm_390.x 16725007

[B14] Hayes D Jr Mycobacterium abscessus and other nontuberculous mycobacteria: evolving respiratory pathogens in cystic fibrosis: a case report and review Southern Med J 2005 98 657 661 10.1097/01.SMJ.0000163311.70464.91 16004174

[B15] Sanguinetti M Ardito F Fiscarelli E La Sorda M D’argenio P Ricciotti G Fadda G Fatal pulmonary infection due to multidrug-resistant Mycobacterium abscessus in a patient with cystic fibrosis J Clin Microbiol 2001 39 816 819 10.1128/JCM.39.2.816-819.2001 11158161 PMC87830

[B16] Shin JH Lee HK Cho EJ Yu JY Kang YH Targeting the rpoB gene using nested PCR-restriction fragment length polymorphism for identification of nontuberculous mycobacteria in hospital tap water J Microbiol 2008 46 608 614 10.1007/s12275-008-0102-6 19107388

[B17] Huang WC Chiou CS Chen JH Shen GH Molecular epidemiology of Mycobacterium abscessus infections in a subtropical chronic ventilatory setting J Med Microbiol 2010 59 1203 1211 10.1099/jmm.0.020586-0 20616186

[B18] Adékambi T Ben Salah I Khlif M Raoult D Drancourt M Survival of environmental mycobacteria in Acanthamoeba polyphaga Appl Environ Microbiol 2006 72 5974 5981 10.1128/AEM.03075-05 16957218 PMC1563627

[B19] Koh WJ Jeon K Lee NY Kim BJ Kook YH Lee SH Park YK Kim CK Shin SJ Huitt GA Daley CL Kwon OJ Clinical significance of differentiation of Mycobacterium massiliense from Mycobacterium abscessus Am J Respir Crit Care Med 2011 183 405 410 10.1164/rccm.201003-0395OC 20833823

[B20] Leao SC Tortoli E Viana-Niero C Ueki SY Lima KV Lopes ML Yubero J Menendez MC Garcia MJ Characterization of mycobacteria from a major Brazilian outbreak suggests that revision of the taxonomic status of members of the Mycobacterium chelonae-M. abscessus group is needed J Clin Microbiol 2009 47 2691 2698 10.1128/JCM.00808-09 19571015 PMC2738059

[B21] Macheras E Roux AL Bastian S Leão SC Palaci M Sivadon-Tardy V Gutierrez C Richter E Rüsch-Gerdes S Pfyffer G Bodmer T Cambau E Gaillard JL Heym B Multilocus sequence analysis and rpoB sequencing of Mycobacterium abscessus (sensu lato) strains J Clin Microbiol 2011 49 491 499 10.1128/JCM.01274-10 21106786 PMC3043527

[B22] Adékambi T Reynaud-Gaubert M Greub G Gevaudan MJ La Scola B Raoult D Drancourt M Amoebal coculture of “mycobacterium massiliense” sp. nov. From the sputum of a patient with hemoptoic pneumonia J Clin Microbiol 2004 42 5493 5501 10.1128/JCM.42.12.5493-5501.2004 15583272 PMC535245

[B23] Adékambi T Drancourt M Dissection of phylogenetic relationships among 19 rapidly growing Mycobacterium species by 16S rRNA, hsp65, sodA, recA and rpoB gene sequencing Int J Syst Evol Microbiol 2004 54 2095 2105 10.1099/ijs.0.63094-0 15545441

[B24] Adékambi T Berger P Raoult D Drancourt M rpoB gene sequence-based characterization of emerging non-tuberculous mycobacteria with descriptions of Mycobacterium bolletii sp. nov., Mycobacterium phocaicum sp. nov. and Mycobacterium aubagnense sp. nov Int J Syst Evol Microbiol 2006 56 133 143 10.1099/ijs.0.63969-0 16403878

[B25] Macheras E Roux AL Ripoll F Sivadon-Tardy V Gutierrez C Gaillard JL Heym B Inaccuracy of single-target sequencing for discriminating species of the Mycobacterium abscessus group J Clin Microbiol 2009 47 2596 2600 10.1128/JCM.00037-09 19515839 PMC2725694

[B26] Cayrou C Turenne C Behr MA Drancourt M Genotyping of Mycobacterium avium complex organisms using multispacer sequence typing Microbiol 2010 156 687 694 10.1099/mic.0.033522-0 19926652

[B27] Djelouadji Z Arnold C Gharbia S Raoult D Drancourt M Multispacer sequence typing for Mycobacterium tuberculosis genotyping PLoS One 2008 3 e2433 10.1371/journal.pone.0002433 18560597 PMC2413405

[B28] Drancourt M Roux V Dang LV Tran-Hung L Castex D Chenal-Francisque V Ogata H Fournier PE Crubézy E Raoult D Genotyping, Orientalis-like Yersinia pestis, and Plague Pandemics Emer Infect Dis 2004 10 1585 1592 10.3201/eid1009.030933 PMC3320270 15498160

[B29] Wenjun LI Mouffok N Rovery C Parola P Raoult D Genotyping Rickettsia conorii detected in patients with Mediterranean spotted fever in Algeria using multispacer typing (MST) Clin Microbiol Inf 2009 15 281 283 10.1111/j.1469-0691.2008.02236.x 19548993

[B30] Foucault C La Scola B Lindroos H Andersson SGE Raoult D Multispacer typing technique for sequence-based typing of Bartonella Quintana J Clin Microbiol 2005 43 41 48 10.1128/JCM.43.1.41-48.2005 15634949 PMC540158

[B31] Hunter PR Gaston MA Numerical index of the discriminatory ability of typing systems: an application of Simpson’s index of diversity J Clin Microbiol 1988 26 2465 2466 3069867 10.1128/jcm.26.11.2465-2466.1988 PMC266921

[B32] Kumar S Tamura K Jakobsen IB Nei M MEGA2: molecular evolutionary genetics analysis software Bioinformatics 2001 17 1244 1245 10.1093/bioinformatics/17.12.1244 11751241

[B33] Zhang Y Rajagopalan M Brown BA Wallace RJ Jr Randomly amplified polymorphic DNA PCR for comparison of Mycobacterium abscessus strains from nosocomial outbreaks J Clin Microbiol 1997 35 3132 3139 9399508 10.1128/jcm.35.12.3132-3139.1997 PMC230136

[B34] Choi GE Chulhun LC Whang J Kim HJ Kwon OJ Koh WJ Shin SJ Efficient differentiation of mycobacterium abscessus complex isolates to the species level by a novel PCR-based variable-number tandem-repeat assay J Clin Microbiol 2011 49 1107 1109 10.1128/JCM.02318-10 21177894 PMC3067748

[B35] Zelazny AM Root JM Shea YR Colombo RE Shamputa IC Stock F Conlan S McNulty S Brown-Elliott BA Wallace RJ Jr Olivier KN Holland SM Sampaio EP Cohort study of molecular identification and typing of Mycobacterium abscessus, Mycobacterium massiliense, and Mycobacterium bolletii J Clin Microbiol 2009 47 1985 1995 10.1128/JCM.01688-08 19420162 PMC2708513

