# Supplementary material for: Mycobacterium abscessus multispacer sequence typing
Source: BMC Microbiol. 2013 Jan 7;13:3. doi: 10.1186/1471-2180-13-3 (PMC3564728; doi:10.1186/1471-2180-13-3)
Supplement: Additional file 1 — rpoB and MLSA genes accession Number of 49 sequenced genomes. [file 1471-2180-13-3-S1.doc]

| **Isolates** | ***rpoB*** | ***argH*** | ***Cya*** | ***murC*** | ***pta*** | ***purH*** |
| --- | --- | --- | --- | --- | --- | --- |
| ***M.abscessus_*ATCC19977_CIP104536T** | [CU458896](http://www.ncbi.nlm.nih.gov/nuccore/CU458896) | [CU458896](http://www.ncbi.nlm.nih.gov/nuccore/CU458896) | [CU458896](http://www.ncbi.nlm.nih.gov/nuccore/CU458896) | [CU458896](http://www.ncbi.nlm.nih.gov/nuccore/CU458896) | [CU458896](http://www.ncbi.nlm.nih.gov/nuccore/CU458896) | [CU458896](http://www.ncbi.nlm.nih.gov/nuccore/CU458896) |
| ***M.abscessus_3A-0930-R_3A_0930_R*** | [gb|AKVB01000011.1|](http://www.ncbi.nlm.nih.gov/nucleotide/392218494?report=genbank&log$=nuclalign&blast_rank=6&RID=7G22V81A01N) | [gb|AKVB01000007.1|](http://www.ncbi.nlm.nih.gov/nucleotide/392228391?report=genbank&log$=nuclalign&blast_rank=3&RID=7G3H3JTH016) | [gb|AKVB01000002.1|](http://www.ncbi.nlm.nih.gov/nucleotide/392232923?report=genbank&log$=nuclalign&blast_rank=3&RID=7G4PE5P701N) | [gb|AKVB01000006.1|](http://www.ncbi.nlm.nih.gov/nucleotide/392230737?report=genbank&log$=nuclalign&blast_rank=2&RID=7G5VA7M901N) | [gb|AKVB01000011.1|](http://www.ncbi.nlm.nih.gov/nucleotide/392218494?report=genbank&log$=nuclalign&blast_rank=3&RID=7G6ZGW3E014) | [gb|AKVB01000003.1|](http://www.ncbi.nlm.nih.gov/nucleotide/392232232?report=genbank&log$=nuclalign&blast_rank=2&RID=7G976GMH014) |
| ***M.abscessus_3A-0930-S_3A_0930_S*** | [gb|AKVC01000010.1|](http://www.ncbi.nlm.nih.gov/nucleotide/392219822?report=genbank&log$=nuclalign&blast_rank=5&RID=7G22V81A01N) | [gb|AKVC01000005.1|](http://www.ncbi.nlm.nih.gov/nucleotide/392229441?report=genbank&log$=nuclalign&blast_rank=2&RID=7G3H3JTH016) | [gb|AKVC01000006.1|](http://www.ncbi.nlm.nih.gov/nucleotide/392227517?report=genbank&log$=nuclalign&blast_rank=4&RID=7G4PE5P701N) | [gb|AKVC01000005.1|](http://www.ncbi.nlm.nih.gov/nucleotide/392229441?report=genbank&log$=nuclalign&blast_rank=3&RID=7G5VA7M901N) | [gb|AKVC01000010.1|](http://www.ncbi.nlm.nih.gov/nucleotide/392219822?report=genbank&log$=nuclalign&blast_rank=2&RID=7G6ZGW3E014) | [gb|AKVC01000004.1|](http://www.ncbi.nlm.nih.gov/nucleotide/392231522?report=genbank&log$=nuclalign&blast_rank=3&RID=7G976GMH014) |
| ***M.abscessus_3A-0122-S_3A_0122_S*** | [gb|AKUZ01000017.1|](http://www.ncbi.nlm.nih.gov/nucleotide/392205353?report=genbank&log$=nuclalign&blast_rank=8&RID=7G22V81A01N) | [gb|AKUZ01000013.1|](http://www.ncbi.nlm.nih.gov/nucleotide/392210432?report=genbank&log$=nuclalign&blast_rank=5&RID=7G3H3JTH016) | [gb|AKUZ01000002.1|](http://www.ncbi.nlm.nih.gov/nucleotide/392217474?report=genbank&log$=nuclalign&blast_rank=6&RID=7G4PE5P701N) | [gb|AKUZ01000012.1|](http://www.ncbi.nlm.nih.gov/nucleotide/392212325?report=genbank&log$=nuclalign&blast_rank=5&RID=7G5VA7M901N) | [gb|AKUZ01000017.1|](http://www.ncbi.nlm.nih.gov/nucleotide/392205353?report=genbank&log$=nuclalign&blast_rank=5&RID=7G6ZGW3E014) | [gb|AKUZ01000004.1|](http://www.ncbi.nlm.nih.gov/nucleotide/392216803?report=genbank&log$=nuclalign&blast_rank=4&RID=7G976GMH014) |
| ***M.abscessus_3A-0731_3A_0731*** | [gb|AKVA01000011.1|](http://www.ncbi.nlm.nih.gov/nucleotide/392208749?report=genbank&log$=nuclalign&blast_rank=7&RID=7G22V81A01N) | [gb|AKVA01000007.1|](http://www.ncbi.nlm.nih.gov/nucleotide/392214118?report=genbank&log$=nuclalign&blast_rank=4&RID=7G3H3JTH016) | [gb|AKVA01000006.1|](http://www.ncbi.nlm.nih.gov/nucleotide/392214930?report=genbank&log$=nuclalign&blast_rank=7&RID=7G4PE5P701N) | [gb|AKVA01000006.1|](http://www.ncbi.nlm.nih.gov/nucleotide/392214930?report=genbank&log$=nuclalign&blast_rank=4&RID=7G5VA7M901N) | [gb|AKVA01000011.1|](http://www.ncbi.nlm.nih.gov/nucleotide/392208749?report=genbank&log$=nuclalign&blast_rank=4&RID=7G6ZGW3E014) | [gb|AKVA01000005.1|](http://www.ncbi.nlm.nih.gov/nucleotide/392216216?report=genbank&log$=nuclalign&blast_rank=5&RID=7G976GMH014) |
| ***M.abscessus_3A-0122-R_3A_0122_R*** | [gb|AKUY01000032.1|](http://www.ncbi.nlm.nih.gov/nucleotide/392197599?report=genbank&log$=nuclalign&blast_rank=9&RID=7G22V81A01N) | [gb|AKUY01000022.1|](http://www.ncbi.nlm.nih.gov/nucleotide/392204618?report=genbank&log$=nuclalign&blast_rank=6&RID=7G3H3JTH016) | [gb|AKUY01000007.1|](http://www.ncbi.nlm.nih.gov/nucleotide/392213735?report=genbank&log$=nuclalign&blast_rank=8&RID=7G4PE5P701N) | [gb|AKUY01000020.1|](http://www.ncbi.nlm.nih.gov/nucleotide/392205021?report=genbank&log$=nuclalign&blast_rank=6&RID=7G5VA7M901N) | [gb|AKUY01000032.1|](http://www.ncbi.nlm.nih.gov/nucleotide/392197599?report=genbank&log$=nuclalign&blast_rank=6&RID=7G6ZGW3E014) | [gb|AKUY01000029.1|](http://www.ncbi.nlm.nih.gov/nucleotide/392200670?report=genbank&log$=nuclalign&blast_rank=7&RID=7G976GMH014) |
| ***M.abscessus_3A-0119-R_3A_0119_R*** | [gb|AKUX01000012.1|](http://www.ncbi.nlm.nih.gov/nucleotide/392194630?report=genbank&log$=nuclalign&blast_rank=10&RID=7G22V81A01N) | [gb|AKUX01000010.1|](http://www.ncbi.nlm.nih.gov/nucleotide/392198804?report=genbank&log$=nuclalign&blast_rank=7&RID=7G3H3JTH016) | [gb|AKUX01000002.1|](http://www.ncbi.nlm.nih.gov/nucleotide/392204696?report=genbank&log$=nuclalign&blast_rank=9&RID=7G4PE5P701N) | [gb|AKUX01000009.1|](http://www.ncbi.nlm.nih.gov/nucleotide/392199951?report=genbank&log$=nuclalign&blast_rank=7&RID=7G5VA7M901N) | [gb|AKUX01000012.1|](http://www.ncbi.nlm.nih.gov/nucleotide/392194630?report=genbank&log$=nuclalign&blast_rank=7&RID=7G6ZGW3E014) | [gb|AKUX01000005.1|](http://www.ncbi.nlm.nih.gov/nucleotide/392203308?report=genbank&log$=nuclalign&blast_rank=6&RID=7G976GMH014) |
| ***M.abscessus_6G-0728-R_M6G_0728_R*** | [gb|AKUS01000008.1|](http://www.ncbi.nlm.nih.gov/nucleotide/392168450?report=genbank&log$=nuclalign&blast_rank=12&RID=7G22V81A01N) | [gb|AKUS01000006.1|](http://www.ncbi.nlm.nih.gov/nucleotide/392172297?report=genbank&log$=nuclalign&blast_rank=8&RID=7G3H3JTH016) | [gb|AKUS01000002.1|](http://www.ncbi.nlm.nih.gov/nucleotide/392176354?report=genbank&log$=nuclalign&blast_rank=11&RID=7G4PE5P701N) | [gb|AKUS01000005.1|](http://www.ncbi.nlm.nih.gov/nucleotide/392173883?report=genbank&log$=nuclalign&blast_rank=8&RID=7G5VA7M901N) | [gb|AKUS01000008.1|](http://www.ncbi.nlm.nih.gov/nucleotide/392168450?report=genbank&log$=nuclalign&blast_rank=8&RID=7G6ZGW3E014) | [gb|AKUS01000003.1|](http://www.ncbi.nlm.nih.gov/nucleotide/392174905?report=genbank&log$=nuclalign&blast_rank=8&RID=7G976GMH014) |
| ***M.abscessus_6G-0212_M6G_0212*** | [gb|AKUR01000012.1|](http://www.ncbi.nlm.nih.gov/nucleotide/392162643?report=genbank&log$=nuclalign&blast_rank=13&RID=7G22V81A01N) | [gb|AKUR01000010.1|](http://www.ncbi.nlm.nih.gov/nucleotide/392166640?report=genbank&log$=nuclalign&blast_rank=9&RID=7G3H3JTH016) | [gb|AKUR01000003.1|](http://www.ncbi.nlm.nih.gov/nucleotide/392173488?report=genbank&log$=nuclalign&blast_rank=12&RID=7G4PE5P701N) | [gb|AKUR01000009.1|](http://www.ncbi.nlm.nih.gov/nucleotide/392167758?report=genbank&log$=nuclalign&blast_rank=9&RID=7G5VA7M901N) | [gb|AKUR01000012.1|](http://www.ncbi.nlm.nih.gov/nucleotide/392162643?report=genbank&log$=nuclalign&blast_rank=9&RID=7G6ZGW3E014) | [gb|AKUR01000005.1|](http://www.ncbi.nlm.nih.gov/nucleotide/392171967?report=genbank&log$=nuclalign&blast_rank=9&RID=7G976GMH014) |
| ***M.abscessus _6G-1108_6G_1108*** | [gb|AKUH01000015.1|](http://www.ncbi.nlm.nih.gov/nucleotide/392129228?report=genbank&log$=nuclalign&blast_rank=14&RID=7G22V81A01N) | [gb|AKUH01000013.1|](http://www.ncbi.nlm.nih.gov/nucleotide/392134333?report=genbank&log$=nuclalign&blast_rank=10&RID=7G3H3JTH016) | [gb|AKUH01000006.1|](http://www.ncbi.nlm.nih.gov/nucleotide/392140851?report=genbank&log$=nuclalign&blast_rank=13&RID=7G4PE5P701N) | [gb|AKUH01000012.1|](http://www.ncbi.nlm.nih.gov/nucleotide/392135518?report=genbank&log$=nuclalign&blast_rank=14&RID=7G5VA7M901N) | [gb|AKUH01000016.1|](http://www.ncbi.nlm.nih.gov/nucleotide/392126034?report=genbank&log$=nuclalign&blast_rank=11&RID=7G6ZGW3E014) | [gb|AKUH01000008.1|](http://www.ncbi.nlm.nih.gov/nucleotide/392140301?report=genbank&log$=nuclalign&blast_rank=10&RID=7G976GMH014) |
| ***M.abscessus _6G-0728-S_6G_0728_S*** | [gb|AKUG01000006.1|](http://www.ncbi.nlm.nih.gov/nucleotide/392127015?report=genbank&log$=nuclalign&blast_rank=15&RID=7G22V81A01N) | [gb|AKUG01000005.1|](http://www.ncbi.nlm.nih.gov/nucleotide/392130164?report=genbank&log$=nuclalign&blast_rank=11&RID=7G3H3JTH016) | [gb|AKUG01000003.1|](http://www.ncbi.nlm.nih.gov/nucleotide/392135257?report=genbank&log$=nuclalign&blast_rank=14&RID=7G4PE5P701N) | [gb|AKUG01000006.1|](http://www.ncbi.nlm.nih.gov/nucleotide/392127015?report=genbank&log$=nuclalign&blast_rank=15&RID=7G5VA7M901N) | [gb|AKUG01000006.1|](http://www.ncbi.nlm.nih.gov/nucleotide/392127015?report=genbank&log$=nuclalign&blast_rank=10&RID=7G6ZGW3E014) | [gb|AKUG01000005.1|](http://www.ncbi.nlm.nih.gov/nucleotide/392130164?report=genbank&log$=nuclalign&blast_rank=11&RID=7G976GMH014) |
| ***M.abscessus_6G-0125-R_6G_0125_R*** | [gb|AKUE01000005.1|](http://www.ncbi.nlm.nih.gov/nucleotide/392112025?report=genbank&log$=nuclalign&blast_rank=16&RID=7G22V81A01N) | [gb|AKUE01000004.1|](http://www.ncbi.nlm.nih.gov/nucleotide/392121337?report=genbank&log$=nuclalign&blast_rank=12&RID=7G3H3JTH016) | [b|AKUE01000005.1|](http://www.ncbi.nlm.nih.gov/nucleotide/392112025?report=genbank&log$=nuclalign&blast_rank=16&RID=7G4PE5P701N) | [gb|AKUE01000004.1|](http://www.ncbi.nlm.nih.gov/nucleotide/392121337?report=genbank&log$=nuclalign&blast_rank=17&RID=7G5VA7M901N) | [gb|AKUE01000005.1|](http://www.ncbi.nlm.nih.gov/nucleotide/392112025?report=genbank&log$=nuclalign&blast_rank=12&RID=7G6ZGW3E014) | [gb|AKUE01000001.1|](http://www.ncbi.nlm.nih.gov/nucleotide/392124967?report=genbank&log$=nuclalign&blast_rank=12&RID=7G976GMH014) |
| ***M.abscessus _6G-0125-S_6G_0125_S*** | [gb|AKUF01000008.1|](http://www.ncbi.nlm.nih.gov/nucleotide/392110088?report=genbank&log$=nuclalign&blast_rank=17&RID=7G22V81A01N) | [gb|AKUF01000007.1|](http://www.ncbi.nlm.nih.gov/nucleotide/392120352?report=genbank&log$=nuclalign&blast_rank=13&RID=7G3H3JTH016) | [gb|AKUF01000004.1|](http://www.ncbi.nlm.nih.gov/nucleotide/392125299?report=genbank&log$=nuclalign&blast_rank=15&RID=7G4PE5P701N) | [gb|AKUF01000006.1|](http://www.ncbi.nlm.nih.gov/nucleotide/392122577?report=genbank&log$=nuclalign&blast_rank=16&RID=7G5VA7M901N) | [gb|AKUF01000008.1|](http://www.ncbi.nlm.nih.gov/nucleotide/392110088?report=genbank&log$=nuclalign&blast_rank=13&RID=7G6ZGW3E014) | [gb|AKUF01000006.1|](http://www.ncbi.nlm.nih.gov/nucleotide/392122577?report=genbank&log$=nuclalign&blast_rank=13&RID=7G976GMH014) |
| ***M.abscessus_4S-0116-S_4S_0116_S*** | [gb|AKVE01000007.1|](http://www.ncbi.nlm.nih.gov/nucleotide/392233575?report=genbank&log$=nuclalign&blast_rank=3&RID=7G22V81A01N) | [gb|AKVE01000006.1|](http://www.ncbi.nlm.nih.gov/nucleotide/392236882?report=genbank&log$=nuclalign&blast_rank=15&RID=7G3H3JTH016) | [gb|AKVE01000007.1|](http://www.ncbi.nlm.nih.gov/nucleotide/392233575?report=genbank&log$=nuclalign&blast_rank=2&RID=7G4PE5P701N) | [gb|AKVE01000006.1|](http://www.ncbi.nlm.nih.gov/nucleotide/392236882?report=genbank&log$=nuclalign&blast_rank=18&RID=7G5VA7M901N) | [gb|AKVE01000007.1|](http://www.ncbi.nlm.nih.gov/nucleotide/392233575?report=genbank&log$=nuclalign&blast_rank=17&RID=7G6ZGW3E014) | [gb|AKVE01000001.1|](http://www.ncbi.nlm.nih.gov/nucleotide/392243181?report=genbank&log$=nuclalign&blast_rank=15&RID=7G976GMH014) |
| ***M.abscessus_4S-0116-R_4S_0116_R*** | [gb|AKVD01000006.1|](http://www.ncbi.nlm.nih.gov/nucleotide/392221153?report=genbank&log$=nuclalign&blast_rank=4&RID=7G22V81A01N) | [gb|AKVD01000002.1|](http://www.ncbi.nlm.nih.gov/nucleotide/392229069?report=genbank&log$=nuclalign&blast_rank=16&RID=7G3H3JTH016) | [gb|AKVD01000003.1|](http://www.ncbi.nlm.nih.gov/nucleotide/392225974?report=genbank&log$=nuclalign&blast_rank=5&RID=7G4PE5P701N) | [gb|AKVD01000004.1|](http://www.ncbi.nlm.nih.gov/nucleotide/392225412?report=genbank&log$=nuclalign&blast_rank=19&RID=7G5VA7M901N) | [gb|AKVD01000006.1|](http://www.ncbi.nlm.nih.gov/nucleotide/392221153?report=genbank&log$=nuclalign&blast_rank=18&RID=7G6ZGW3E014) | [gb|AKVD01000003.1|](http://www.ncbi.nlm.nih.gov/nucleotide/392225974?report=genbank&log$=nuclalign&blast_rank=16&RID=7G976GMH014) |
| ***M.abscessus_4S-0206_M4S_0206*** | [gb|AKUT01000010.1|](http://www.ncbi.nlm.nih.gov/nucleotide/392181472?report=genbank&log$=nuclalign&blast_rank=11&RID=7G22V81A01N) | [gb|AKUT01000010.1|](http://www.ncbi.nlm.nih.gov/nucleotide/392181472?report=genbank&log$=nuclalign&blast_rank=17&RID=7G3H3JTH016) | [gb|AKUT01000010.1|](http://www.ncbi.nlm.nih.gov/nucleotide/392181472?report=genbank&log$=nuclalign&blast_rank=10&RID=7G4PE5P701N) | [gb|AKUT01000008.1|](http://www.ncbi.nlm.nih.gov/nucleotide/392186745?report=genbank&log$=nuclalign&blast_rank=20&RID=7G5VA7M901N) | [gb|AKUT01000010.1|](http://www.ncbi.nlm.nih.gov/nucleotide/392181472?report=genbank&log$=nuclalign&blast_rank=19&RID=7G6ZGW3E014) | [gb|AKUT01000004.1|](http://www.ncbi.nlm.nih.gov/nucleotide/392188406?report=genbank&log$=nuclalign&blast_rank=17&RID=7G976GMH014) |
| ***M.abscessus_4S-0726-RB_4S_0726_RB*** | [gb|AKTW01000005.1|](http://www.ncbi.nlm.nih.gov/nucleotide/392065242?report=genbank&log$=nuclalign&blast_rank=19&RID=7G22V81A01N) | [gb|AKTW01000004.1|](http://www.ncbi.nlm.nih.gov/nucleotide/392071901?report=genbank&log$=nuclalign&blast_rank=19&RID=7G3H3JTH016) | [gb|AKTW01000005.1|](http://www.ncbi.nlm.nih.gov/nucleotide/392065242?report=genbank&log$=nuclalign&blast_rank=19&RID=7G4PE5P701N) | [gb|AKTW01000003.1|](http://www.ncbi.nlm.nih.gov/nucleotide/392073314?report=genbank&log$=nuclalign&blast_rank=23&RID=7G5VA7M901N) | [gb|AKTW01000005.1|](http://www.ncbi.nlm.nih.gov/nucleotide/392065242?report=genbank&log$=nuclalign&blast_rank=21&RID=7G6ZGW3E014) | [gb|AKTW01000001.1|](http://www.ncbi.nlm.nih.gov/nucleotide/392075568?report=genbank&log$=nuclalign&blast_rank=18&RID=7G976GMH014) |
| ***M.abscessus_4S-0303_4S_0303*** | [gb|AKTU01000008.1|](http://www.ncbi.nlm.nih.gov/nucleotide/392063248?report=genbank&log$=nuclalign&blast_rank=20&RID=7G22V81A01N) | [gb|AKTU01000001.1|](http://www.ncbi.nlm.nih.gov/nucleotide/392076830?report=genbank&log$=nuclalign&blast_rank=18&RID=7G3H3JTH016) | [gb|AKTU01000001.1|](http://www.ncbi.nlm.nih.gov/nucleotide/392076830?report=genbank&log$=nuclalign&blast_rank=17&RID=7G4PE5P701N) | [gb|AKTU01000001.1|](http://www.ncbi.nlm.nih.gov/nucleotide/392076830?report=genbank&log$=nuclalign&blast_rank=21&RID=7G5VA7M901N) | [gb|AKTU01000008.1|](http://www.ncbi.nlm.nih.gov/nucleotide/392063248?report=genbank&log$=nuclalign&blast_rank=22&RID=7G6ZGW3E014) | [gb|AKTU01000004.1|](http://www.ncbi.nlm.nih.gov/nucleotide/392073515?report=genbank&log$=nuclalign&blast_rank=19&RID=7G976GMH014) |
| ***M.abscessus_4S-0726-RA_4S_0726_RA*** | [gb|AKTV01000006.1|](http://www.ncbi.nlm.nih.gov/nucleotide/392067710?report=genbank&log$=nuclalign&blast_rank=18&RID=7G22V81A01N) | [gb|AKTV01000006.1|](http://www.ncbi.nlm.nih.gov/nucleotide/392067710?report=genbank&log$=nuclalign&blast_rank=20&RID=7G3H3JTH016) | g[b|AKTV01000001.1|](http://www.ncbi.nlm.nih.gov/nucleotide/392074858?report=genbank&log$=nuclalign&blast_rank=18&RID=7G4PE5P701N) | [gb|AKTV01000001.1|](http://www.ncbi.nlm.nih.gov/nucleotide/392074858?report=genbank&log$=nuclalign&blast_rank=22&RID=7G5VA7M901N) | [gb|AKTV01000006.1|](http://www.ncbi.nlm.nih.gov/nucleotide/392067710?report=genbank&log$=nuclalign&blast_rank=20&RID=7G6ZGW3E014) | [gb|AKTV01000003.1|](http://www.ncbi.nlm.nih.gov/nucleotide/392072795?report=genbank&log$=nuclalign&blast_rank=20&RID=7G976GMH014) |
| ***M.abscessus_M93*** | [gb|AJGF01000005.1|](http://www.ncbi.nlm.nih.gov/nucleotide/382942555?report=genbank&log$=nuclalign&blast_rank=22&RID=7G22V81A01N) | [gb|AJGF01000001.1|](http://www.ncbi.nlm.nih.gov/nucleotide/382944919?report=genbank&log$=nuclalign&blast_rank=21&RID=7G3H3JTH016) | [gb|AJGF01000009.1|](http://www.ncbi.nlm.nih.gov/nucleotide/382939680?report=genbank&log$=nuclalign&blast_rank=20&RID=7G4PE5P701N) | [gb|AJGF01000004.1|](http://www.ncbi.nlm.nih.gov/nucleotide/382943748?report=genbank&log$=nuclalign&blast_rank=25&RID=7G5VA7M901N) | [gb|AJGF01000013.1|](http://www.ncbi.nlm.nih.gov/nucleotide/382937632?report=genbank&log$=nuclalign&blast_rank=15&RID=7G6ZGW3E014) | [gb|AJGF01000008.1|](http://www.ncbi.nlm.nih.gov/nucleotide/382940451?report=genbank&log$=nuclalign&blast_rank=21&RID=7G976GMH014) |
| ***M.abscessus_M94*** | [gb|AJGG01000030.1|](http://www.ncbi.nlm.nih.gov/nucleotide/382942156?report=genbank&log$=nuclalign&blast_rank=21&RID=7G22V81A01N) | [gb|AJGG01000029.1|](http://www.ncbi.nlm.nih.gov/nucleotide/382942418?report=genbank&log$=nuclalign&blast_rank=23&RID=7G3H3JTH016) | [gb|AJGG01000006.1|](http://www.ncbi.nlm.nih.gov/nucleotide/382946247?report=genbank&log$=nuclalign&blast_rank=22&RID=7G4PE5P701N) | [gb|AJGG01000003.1|](http://www.ncbi.nlm.nih.gov/nucleotide/382946585?report=genbank&log$=nuclalign&blast_rank=24&RID=7G5VA7M901N) | [gb|AJGG01000001.1|](http://www.ncbi.nlm.nih.gov/nucleotide/382946870?report=genbank&log$=nuclalign&blast_rank=14&RID=7G6ZGW3E014) | [gb|AJGG01000018.1|](http://www.ncbi.nlm.nih.gov/nucleotide/382945242?report=genbank&log$=nuclalign&blast_rank=22&RID=7G976GMH014) |
| ***M.abscessus_M152*** | [gb|AKVT01000004.1|](http://www.ncbi.nlm.nih.gov/nucleotide/396586800?report=genbank&log$=nuclalign&blast_rank=1&RID=7FSHDB5C01N) | [gb|AKVT01000015.1|](http://www.ncbi.nlm.nih.gov/nucleotide/396586789?report=genbank&log$=nuclalign&blast_rank=22&RID=7G3H3JTH016) | [gb|AKVT01000001.1|](http://www.ncbi.nlm.nih.gov/nucleotide/396586803?report=genbank&log$=nuclalign&blast_rank=21&RID=7G4PE5P701N) | [gb|AKVT01000002.1|](http://www.ncbi.nlm.nih.gov/nucleotide/396586802?report=genbank&log$=nuclalign&blast_rank=26&RID=7G5VA7M901N) | [gb|AKVT01000006.1|](http://www.ncbi.nlm.nih.gov/nucleotide/396586798?report=genbank&log$=nuclalign&blast_rank=16&RID=7G6ZGW3E014) | [gb|AKVT01000016.1|](http://www.ncbi.nlm.nih.gov/nucleotide/396586788?report=genbank&log$=nuclalign&blast_rank=14&RID=7G976GMH014) |
| ***M.bolletti_*CIP108541T** | [gb|AHAS01000016.1|](http://www.ncbi.nlm.nih.gov/nucleotide/363995452?report=genbank&log$=nuclalign&blast_rank=53&RID=7G22V81A01N) | [gb|AHAS01000010.1|](http://www.ncbi.nlm.nih.gov/nucleotide/363999164?report=genbank&log$=nuclalign&blast_rank=25&RID=7G3H3JTH016) | [gb|AHAS01000004.1|](http://www.ncbi.nlm.nih.gov/nucleotide/364001619?report=genbank&log$=nuclalign&blast_rank=27&RID=7G4PE5P701N) | [gb|AHAS01000008.1|](http://www.ncbi.nlm.nih.gov/nucleotide/363999800?report=genbank&log$=nuclalign&blast_rank=53&RID=7G5VA7M901N) | [gb|AHAS01000016.1|](http://www.ncbi.nlm.nih.gov/nucleotide/363995452?report=genbank&log$=nuclalign&blast_rank=34&RID=7G6ZGW3E014) | [gb|AHAS01000006.1|](http://www.ncbi.nlm.nih.gov/nucleotide/364000832?report=genbank&log$=nuclalign&blast_rank=26&RID=7G976GMH014) |
| ***M.abscessus_M24*** | [gb|AJLY01000081.1|](http://www.ncbi.nlm.nih.gov/nucleotide/385692619?report=genbank&log$=nuclalign&blast_rank=52&RID=7G22V81A01N) | [gb|AJLY01000082.1|](http://www.ncbi.nlm.nih.gov/nucleotide/385692618?report=genbank&log$=nuclalign&blast_rank=24&RID=7G3H3JTH016) | [gb|AJLY01000009.1|](http://www.ncbi.nlm.nih.gov/nucleotide/385692691?report=genbank&log$=nuclalign&blast_rank=53&RID=7G4PE5P701N) | [gb|AJLY01000046.1|](http://www.ncbi.nlm.nih.gov/nucleotide/385692654?report=genbank&log$=nuclalign&blast_rank=54&RID=7G5VA7M901N) | [gb|AJLY01000039.1|](http://www.ncbi.nlm.nih.gov/nucleotide/385692661?report=genbank&log$=nuclalign&blast_rank=24&RID=7G6ZGW3E014) | [gb|AJLY01000165.1|](http://www.ncbi.nlm.nih.gov/nucleotide/385692535?report=genbank&log$=nuclalign&blast_rank=23&RID=7G976GMH014) |
| ***M.massilliense_*CIP108297T** | [gb|AHAR01000015.1|](http://www.ncbi.nlm.nih.gov/nucleotide/363993997?report=genbank&log$=nuclalign&blast_rank=46&RID=7G22V81A01N) | [gb|AHAR01000010.1|](http://www.ncbi.nlm.nih.gov/nucleotide/363997224?report=genbank&log$=nuclalign&blast_rank=51&RID=7G3H3JTH016) | [gb|AHAR01000003.1|](http://www.ncbi.nlm.nih.gov/nucleotide/364000509?report=genbank&log$=nuclalign&blast_rank=51&RID=7G4PE5P701N) | [gb|AHAR01000009.1|](http://www.ncbi.nlm.nih.gov/nucleotide/363997721?report=genbank&log$=nuclalign&blast_rank=48&RID=7G5VA7M901N) | [gb|AHAR01000015.1|](http://www.ncbi.nlm.nih.gov/nucleotide/363993997?report=genbank&log$=nuclalign&blast_rank=35&RID=7G6ZGW3E014) | [gb|AHAR01000007.1|](http://www.ncbi.nlm.nih.gov/nucleotide/363998470?report=genbank&log$=nuclalign&blast_rank=27&RID=7G976GMH014) |
| ***M.massiliense _2B-0912-S_2B_0912_S*** | [gb|AKUW01000012.1|](http://www.ncbi.nlm.nih.gov/nucleotide/392191999?report=genbank&log$=nuclalign&blast_rank=27&RID=7G22V81A01N) | [gb|AKUW01000008.1|](http://www.ncbi.nlm.nih.gov/nucleotide/392197172?report=genbank&log$=nuclalign&blast_rank=31&RID=7G3H3JTH016) | [gb|AKUW01000001.1|](http://www.ncbi.nlm.nih.gov/nucleotide/392202357?report=genbank&log$=nuclalign&blast_rank=30&RID=7G4PE5P701N) | [gb|AKUW01000008.1|](http://www.ncbi.nlm.nih.gov/nucleotide/392197172?report=genbank&log$=nuclalign&blast_rank=33&RID=7G5VA7M901N) | [gb|AKUW01000012.1|](http://www.ncbi.nlm.nih.gov/nucleotide/392191999?report=genbank&log$=nuclalign&blast_rank=38&RID=7G6ZGW3E014) | [gb|AKUW01000002.1|](http://www.ncbi.nlm.nih.gov/nucleotide/392201533?report=genbank&log$=nuclalign&blast_rank=41&RID=7G976GMH014) |
| ***M.massiliense_2B-030_ M2B_0307*** | [gb|AKUU01000008.1|](http://www.ncbi.nlm.nih.gov/nucleotide/392178984?report=genbank&log$=nuclalign&blast_rank=28&RID=7G22V81A01N) | [gb|AKUU01000006.1|](http://www.ncbi.nlm.nih.gov/nucleotide/392186547?report=genbank&log$=nuclalign&blast_rank=33&RID=7G3H3JTH016) | [gb|AKUU01000008.1|](http://www.ncbi.nlm.nih.gov/nucleotide/392178984?report=genbank&log$=nuclalign&blast_rank=32&RID=7G4PE5P701N) | [gb|AKUU01000005.1|](http://www.ncbi.nlm.nih.gov/nucleotide/392186983?report=genbank&log$=nuclalign&blast_rank=35&RID=7G5VA7M901N) | [gb|AKUU01000008.1|](http://www.ncbi.nlm.nih.gov/nucleotide/392178984?report=genbank&log$=nuclalign&blast_rank=39&RID=7G6ZGW3E014) | [gb|AKUU01000002.1|](http://www.ncbi.nlm.nih.gov/nucleotide/392189774?report=genbank&log$=nuclalign&blast_rank=42&RID=7G976GMH014) |
| ***M.massiliense_2B-0912-R_2B_0912_R*** | [gb|AKUV01000009.1|](http://www.ncbi.nlm.nih.gov/nucleotide/392177556?report=genbank&log$=nuclalign&blast_rank=29&RID=7G22V81A01N) | [gb|AKUV01000006.1|](http://www.ncbi.nlm.nih.gov/nucleotide/392187233?report=genbank&log$=nuclalign&blast_rank=32&RID=7G3H3JTH016) | [gb|AKUV01000001.1|](http://www.ncbi.nlm.nih.gov/nucleotide/392191194?report=genbank&log$=nuclalign&blast_rank=31&RID=7G4PE5P701N) | [gb|AKUV01000006.1|](http://www.ncbi.nlm.nih.gov/nucleotide/392187233?report=genbank&log$=nuclalign&blast_rank=34&RID=7G5VA7M901N) | [gb|AKUV01000009.1|](http://www.ncbi.nlm.nih.gov/nucleotide/392177556?report=genbank&log$=nuclalign&blast_rank=40&RID=7G6ZGW3E014) | [gb|AKUV01000004.1|](http://www.ncbi.nlm.nih.gov/nucleotide/392188830?report=genbank&log$=nuclalign&blast_rank=43&RID=7G976GMH014) |
| ***M.massiliense_2B-0626_M2B_0626*** | [gb|AKUM01000006.1|](http://www.ncbi.nlm.nih.gov/nucleotide/392151792?report=genbank&log$=nuclalign&blast_rank=32&RID=7G22V81A01N) | [gb|AKUM01000004.1|](http://www.ncbi.nlm.nih.gov/nucleotide/392157108?report=genbank&log$=nuclalign&blast_rank=35&RID=7G3H3JTH016) | [gb|AKUM01000001.1|](http://www.ncbi.nlm.nih.gov/nucleotide/392164887?report=genbank&log$=nuclalign&blast_rank=34&RID=7G4PE5P701N) | [gb|AKUM01000003.1|](http://www.ncbi.nlm.nih.gov/nucleotide/392159524?report=genbank&log$=nuclalign&blast_rank=37&RID=7G5VA7M901N) | [gb|AKUM01000006.1|](http://www.ncbi.nlm.nih.gov/nucleotide/392151792?report=genbank&log$=nuclalign&blast_rank=42&RID=7G6ZGW3E014) | [gb|AKUM01000002.1|](http://www.ncbi.nlm.nih.gov/nucleotide/392160229?report=genbank&log$=nuclalign&blast_rank=44&RID=7G976GMH014) |
| ***M.massiliense_2B-1231_M2B_1231*** | [gb|AKUO01000009.1|](http://www.ncbi.nlm.nih.gov/nucleotide/392245378?report=genbank&log$=nuclalign&blast_rank=25&RID=7G22V81A01N) | [gb|AKUO01000007.1|](http://www.ncbi.nlm.nih.gov/nucleotide/392251287?report=genbank&log$=nuclalign&blast_rank=29&RID=7G3H3JTH016) | [gb|AKUO01000002.1|](http://www.ncbi.nlm.nih.gov/nucleotide/392256715?report=genbank&log$=nuclalign&blast_rank=28&RID=7G4PE5P701N) | [gb|AKUO01000007.1|](http://www.ncbi.nlm.nih.gov/nucleotide/392251287?report=genbank&log$=nuclalign&blast_rank=30&RID=7G5VA7M901N) | [gb|AKUO01000009.1|](http://www.ncbi.nlm.nih.gov/nucleotide/392245378?report=genbank&log$=nuclalign&blast_rank=37&RID=7G6ZGW3E014) | [gb|AKUO01000004.1|](http://www.ncbi.nlm.nih.gov/nucleotide/392254710?report=genbank&log$=nuclalign&blast_rank=40&RID=7G976GMH014) |
| ***M.massiliense_2B-0107_M2B_0107*** | [gb|AKUN01000007.1|](http://www.ncbi.nlm.nih.gov/nucleotide/392247182?report=genbank&log$=nuclalign&blast_rank=24&RID=7G22V81A01N) | [gb|AKUN01000004.1|](http://www.ncbi.nlm.nih.gov/nucleotide/392252864?report=genbank&log$=nuclalign&blast_rank=28&RID=7G3H3JTH016) | [gb|AKUN01000007.1|](http://www.ncbi.nlm.nih.gov/nucleotide/392247182?report=genbank&log$=nuclalign&blast_rank=29&RID=7G4PE5P701N) | [gb|AKUN01000003.1|](http://www.ncbi.nlm.nih.gov/nucleotide/392254395?report=genbank&log$=nuclalign&blast_rank=29&RID=7G5VA7M901N) | [gb|AKUN01000007.1|](http://www.ncbi.nlm.nih.gov/nucleotide/392247182?report=genbank&log$=nuclalign&blast_rank=36&RID=7G6ZGW3E014) | [gb|AKUN01000002.1|](http://www.ncbi.nlm.nih.gov/nucleotide/392255310?report=genbank&log$=nuclalign&blast_rank=39&RID=7G976GMH014) |
| ***M.massiliense _1S-154-0310_M1S_154_0310*** | [gb|AKUL01000004.1|](http://www.ncbi.nlm.nih.gov/nucleotide/392153533?report=genbank&log$=nuclalign&blast_rank=31&RID=7G22V81A01N) | [gb|AKUL01000004.1|](http://www.ncbi.nlm.nih.gov/nucleotide/392153533?report=genbank&log$=nuclalign&blast_rank=36&RID=7G3H3JTH016) | [gb|AKUL01000001.1|](http://www.ncbi.nlm.nih.gov/nucleotide/392158068?report=genbank&log$=nuclalign&blast_rank=35&RID=7G4PE5P701N) | [gb|AKUL01000001.1|](http://www.ncbi.nlm.nih.gov/nucleotide/392158068?report=genbank&log$=nuclalign&blast_rank=10&RID=7G5VA7M901N) | [gb|AKUL01000004.1|](http://www.ncbi.nlm.nih.gov/nucleotide/392153533?report=genbank&log$=nuclalign&blast_rank=41&RID=7G6ZGW3E014) | [gb|AKUL01000001.1|](http://www.ncbi.nlm.nih.gov/nucleotide/392158068?report=genbank&log$=nuclalign&blast_rank=45&RID=7G976GMH014) |
| ***M.massiliense_1S-152-0914_M1S_152_0914*** | [gb|AKUJ01000002.1|](http://www.ncbi.nlm.nih.gov/nucleotide/392141245?report=genbank&log$=nuclalign&blast_rank=34&RID=7G22V81A01N) | [gb|AKUJ01000002.1|](http://www.ncbi.nlm.nih.gov/nucleotide/392141245?report=genbank&log$=nuclalign&blast_rank=38&RID=7G3H3JTH016) | [gb|AKUJ01000001.1|](http://www.ncbi.nlm.nih.gov/nucleotide/392146474?report=genbank&log$=nuclalign&blast_rank=37&RID=7G4PE5P701N) | [gb|AKUJ01000002.1|](http://www.ncbi.nlm.nih.gov/nucleotide/392141245?report=genbank&log$=nuclalign&blast_rank=12&RID=7G5VA7M901N) | [gb|AKUJ01000002.1|](http://www.ncbi.nlm.nih.gov/nucleotide/392141245?report=genbank&log$=nuclalign&blast_rank=44&RID=7G6ZGW3E014) | [gb|AKUJ01000002.1|](http://www.ncbi.nlm.nih.gov/nucleotide/392141245?report=genbank&log$=nuclalign&blast_rank=47&RID=7G976GMH014) |
| ***M.massiliense_1S-153-0915_M1S_153_0915*** | [gb|AKUK01000012.1|](http://www.ncbi.nlm.nih.gov/nucleotide/392147594?report=genbank&log$=nuclalign&blast_rank=33&RID=7G22V81A01N) | [b|AKUK01000010.1|](http://www.ncbi.nlm.nih.gov/nucleotide/392150841?report=genbank&log$=nuclalign&blast_rank=37&RID=7G3H3JTH016) | [gb|AKUK01000001.1|](http://www.ncbi.nlm.nih.gov/nucleotide/392156766?report=genbank&log$=nuclalign&blast_rank=36&RID=7G4PE5P701N) | [b|AKUK01000009.1|](http://www.ncbi.nlm.nih.gov/nucleotide/392151221?report=genbank&log$=nuclalign&blast_rank=11&RID=7G5VA7M901N) | [gb|AKUK01000012.1|](http://www.ncbi.nlm.nih.gov/nucleotide/392147594?report=genbank&log$=nuclalign&blast_rank=43&RID=7G6ZGW3E014) | [gb|AKUK01000005.1|](http://www.ncbi.nlm.nih.gov/nucleotide/392155913?report=genbank&log$=nuclalign&blast_rank=46&RID=7G976GMH014) |
| ***M.massiliense_1S-151-0930_M1S_151_0930*** | [gb|AKUI01000003.1|](http://www.ncbi.nlm.nih.gov/nucleotide/392135785?report=genbank&log$=nuclalign&blast_rank=35&RID=7G22V81A01N) | [gb|AKUI01000003.1|](http://www.ncbi.nlm.nih.gov/nucleotide/392135785?report=genbank&log$=nuclalign&blast_rank=39&RID=7G3H3JTH016) | [b|AKUI01000005.1|](http://www.ncbi.nlm.nih.gov/nucleotide/392132198?report=genbank&log$=nuclalign&blast_rank=38&RID=7G4PE5P701N) | [gb|AKUI01000003.1|](http://www.ncbi.nlm.nih.gov/nucleotide/392135785?report=genbank&log$=nuclalign&blast_rank=13&RID=7G5VA7M901N) | [gb|AKUI01000003.1|](http://www.ncbi.nlm.nih.gov/nucleotide/392135785?report=genbank&log$=nuclalign&blast_rank=45&RID=7G6ZGW3E014) | [gb|AKUI01000002.1|](http://www.ncbi.nlm.nih.gov/nucleotide/392139207?report=genbank&log$=nuclalign&blast_rank=48&RID=7G976GMH014) |
| ***M.massiliense _M18*** | [gb|AJSC01000003.1|](http://www.ncbi.nlm.nih.gov/nucleotide/386380734?report=genbank&log$=nuclalign&blast_rank=44&RID=7G22V81A01N) | [gb|AJSC01000008.1|](http://www.ncbi.nlm.nih.gov/nucleotide/386380718?report=genbank&log$=nuclalign&blast_rank=48&RID=7G3H3JTH016) | [gb|AJSC01000009.1|](http://www.ncbi.nlm.nih.gov/nucleotide/386380714?report=genbank&log$=nuclalign&blast_rank=46&RID=7G4PE5P701N) | [gb|AJSC01000002.1|](http://www.ncbi.nlm.nih.gov/nucleotide/386380738?report=genbank&log$=nuclalign&blast_rank=45&RID=7G5VA7M901N) | [gb|AJSC01000003.1|](http://www.ncbi.nlm.nih.gov/nucleotide/386380734?report=genbank&log$=nuclalign&blast_rank=46&RID=7G6ZGW3E014) | [gb|AJSC01000011.1|](http://www.ncbi.nlm.nih.gov/nucleotide/386380708?report=genbank&log$=nuclalign&blast_rank=51&RID=7G976GMH014) |
| ***M.abscessus_M159*** | [gb|AJSD01000003.1|](http://www.ncbi.nlm.nih.gov/nucleotide/386380821?report=genbank&log$=nuclalign&blast_rank=43&RID=7G22V81A01N) | [gb|AJSD01000035.1|](http://www.ncbi.nlm.nih.gov/nucleotide/386380789?report=genbank&log$=nuclalign&blast_rank=26&RID=7G3H3JTH016) | [gb|AJSD01000009.1|](http://www.ncbi.nlm.nih.gov/nucleotide/386380815?report=genbank&log$=nuclalign&blast_rank=52&RID=7G4PE5P701N) | [gb|AJSD01000045.1|](http://www.ncbi.nlm.nih.gov/nucleotide/386380779?report=genbank&log$=nuclalign&blast_rank=51&RID=7G5VA7M901N) | [gb|AJSD01000011.1|](http://www.ncbi.nlm.nih.gov/nucleotide/386380813?report=genbank&log$=nuclalign&blast_rank=50&RID=7G6ZGW3E014) | [gb|AJSD01000031.1|](http://www.ncbi.nlm.nih.gov/nucleotide/386380793?report=genbank&log$=nuclalign&blast_rank=49&RID=7G976GMH014) |
| ***M.abscessus_47J26*** | [gb|AGQU01000001.1|](http://www.ncbi.nlm.nih.gov/nucleotide/353451453?report=genbank&log$=nuclalign&blast_rank=47&RID=7G22V81A01N) | [gb|AGQU01000001.1|](http://www.ncbi.nlm.nih.gov/nucleotide/353451453?report=genbank&log$=nuclalign&blast_rank=52&RID=7G3H3JTH016) | [gb|AGQU01000012.1|](http://www.ncbi.nlm.nih.gov/nucleotide/353448390?report=genbank&log$=nuclalign&blast_rank=48&RID=7G4PE5P701N) | [gb|AGQU01000002.1|](http://www.ncbi.nlm.nih.gov/nucleotide/353450373?report=genbank&log$=nuclalign&blast_rank=49&RID=7G5VA7M901N) | [gb|AGQU01000003.1|](http://www.ncbi.nlm.nih.gov/nucleotide/353449913?report=genbank&log$=nuclalign&blast_rank=47&RID=7G6ZGW3E014) | [gb|AGQU01000005.1|](http://www.ncbi.nlm.nih.gov/nucleotide/353449333?report=genbank&log$=nuclalign&blast_rank=53&RID=7G976GMH014) |
| ***M.abscessus_M172*** | [gb|AJSE01000002.1|](http://www.ncbi.nlm.nih.gov/nucleotide/386380732?report=genbank&log$=nuclalign&blast_rank=50&RID=7G22V81A01N) | [gb|AJSE01000001.1|](http://www.ncbi.nlm.nih.gov/nucleotide/386380735?report=genbank&log$=nuclalign&blast_rank=47&RID=7G3H3JTH016) | [gb|AJSE01000007.1|](http://www.ncbi.nlm.nih.gov/nucleotide/386380716?report=genbank&log$=nuclalign&blast_rank=25&RID=7G4PE5P701N) | [gb|AJSE01000006.1|](http://www.ncbi.nlm.nih.gov/nucleotide/386380719?report=genbank&log$=nuclalign&blast_rank=46&RID=7G5VA7M901N) | [gb|AJSE01000002.1|](http://www.ncbi.nlm.nih.gov/nucleotide/386380732?report=genbank&log$=nuclalign&blast_rank=51&RID=7G6ZGW3E014) | [gb|AJSE01000008.1|](http://www.ncbi.nlm.nih.gov/nucleotide/386380713?report=genbank&log$=nuclalign&blast_rank=50&RID=7G976GMH014) |
| ***M.abscessus_M154*** | [gb|AJMA01000002.1|](http://www.ncbi.nlm.nih.gov/nucleotide/385692449?report=genbank&log$=nuclalign&blast_rank=51&RID=7G22V81A01N) | [gb|AJMA01000009.1|](http://www.ncbi.nlm.nih.gov/nucleotide/385692416?report=genbank&log$=nuclalign&blast_rank=50&RID=7G3H3JTH016) | [gb|AJMA01000016.1|](http://www.ncbi.nlm.nih.gov/nucleotide/385692394?report=genbank&log$=nuclalign&blast_rank=26&RID=7G4PE5P701N) | [b|AJMA01000011.1|](http://www.ncbi.nlm.nih.gov/nucleotide/385692410?report=genbank&log$=nuclalign&blast_rank=47&RID=7G5VA7M901N) | [gb|AJMA01000002.1|](http://www.ncbi.nlm.nih.gov/nucleotide/385692449?report=genbank&log$=nuclalign&blast_rank=52&RID=7G6ZGW3E014) | [gb|AJMA01000018.1|](http://www.ncbi.nlm.nih.gov/nucleotide/385692389?report=genbank&log$=nuclalign&blast_rank=52&RID=7G976GMH014) |
| ***M.abscessus_5S-1215_5S_1215*** | [gb|AKUD01000004.1|](http://www.ncbi.nlm.nih.gov/nucleotide/392115680?report=genbank&log$=nuclalign&blast_rank=36&RID=7G22V81A01N) | [gb|AKUD01000003.1|](http://www.ncbi.nlm.nih.gov/nucleotide/392119842?report=genbank&log$=nuclalign&blast_rank=40&RID=7G3H3JTH016) | [b|AKUD01000001.1|](http://www.ncbi.nlm.nih.gov/nucleotide/392123896?report=genbank&log$=nuclalign&blast_rank=39&RID=7G4PE5P701N) | [gb|AKUD01000003.1|](http://www.ncbi.nlm.nih.gov/nucleotide/392119842?report=genbank&log$=nuclalign&blast_rank=38&RID=7G5VA7M901N) | [gb|AKUD01000004.1|](http://www.ncbi.nlm.nih.gov/nucleotide/392115680?report=genbank&log$=nuclalign&blast_rank=27&RID=7G6ZGW3E014) | [gb|AKUD01000004.1|](http://www.ncbi.nlm.nih.gov/nucleotide/392115680?report=genbank&log$=nuclalign&blast_rank=29&RID=7G976GMH014) |
| ***M.abscessus_5S-1212_5S_1212*** | [gb|AKUC01000007.1|](http://www.ncbi.nlm.nih.gov/nucleotide/392102766?report=genbank&log$=nuclalign&blast_rank=37&RID=7G22V81A01N) | [gb|AKUC01000006.1|](http://www.ncbi.nlm.nih.gov/nucleotide/392106932?report=genbank&log$=nuclalign&blast_rank=41&RID=7G3H3JTH016) | [gb|AKUC01000007.1|](http://www.ncbi.nlm.nih.gov/nucleotide/392102766?report=genbank&log$=nuclalign&blast_rank=41&RID=7G4PE5P701N) | [gb|AKUC01000006.1|](http://www.ncbi.nlm.nih.gov/nucleotide/392106932?report=genbank&log$=nuclalign&blast_rank=39&RID=7G5VA7M901N) | [gb|AKUC01000007.1|](http://www.ncbi.nlm.nih.gov/nucleotide/392102766?report=genbank&log$=nuclalign&blast_rank=28&RID=7G6ZGW3E014) | [gb|AKUC01000003.1|](http://www.ncbi.nlm.nih.gov/nucleotide/392109378?report=genbank&log$=nuclalign&blast_rank=30&RID=7G976GMH014) |
| ***M.abscessus_5S-0817_5S_0817*** | [gb|AKUB01000007.1|](http://www.ncbi.nlm.nih.gov/nucleotide/392097083?report=genbank&log$=nuclalign&blast_rank=38&RID=7G22V81A01N) | [gb|AKUB01000004.1|](http://www.ncbi.nlm.nih.gov/nucleotide/392101628?report=genbank&log$=nuclalign&blast_rank=43&RID=7G3H3JTH016) | [gb|AKUB01000007.1|](http://www.ncbi.nlm.nih.gov/nucleotide/392097083?report=genbank&log$=nuclalign&blast_rank=42&RID=7G4PE5P701N) | [gb|AKUB01000003.1|](http://www.ncbi.nlm.nih.gov/nucleotide/392106410?report=genbank&log$=nuclalign&blast_rank=40&RID=7G5VA7M901N) | [gb|AKUB01000007.1|](http://www.ncbi.nlm.nih.gov/nucleotide/392097083?report=genbank&log$=nuclalign&blast_rank=29&RID=7G6ZGW3E014) | [gb|AKUB01000001.1|](http://www.ncbi.nlm.nih.gov/nucleotide/392108795?report=genbank&log$=nuclalign&blast_rank=31&RID=7G976GMH014) |
| ***M.abscessus_5S-0708_5S_0708*** | [gb|AKUA01000006.1|](http://www.ncbi.nlm.nih.gov/nucleotide/392094004?report=genbank&log$=nuclalign&blast_rank=39&RID=7G22V81A01N) | [gb|AKUA01000003.1|](http://www.ncbi.nlm.nih.gov/nucleotide/392101758?report=genbank&log$=nuclalign&blast_rank=42&RID=7G3H3JTH016) | [gb|AKUA01000001.1|](http://www.ncbi.nlm.nih.gov/nucleotide/392108291?report=genbank&log$=nuclalign&blast_rank=40&RID=7G4PE5P701N) | [gb|AKUA01000003.1|](http://www.ncbi.nlm.nih.gov/nucleotide/392101758?report=genbank&log$=nuclalign&blast_rank=41&RID=7G5VA7M901N) | [gb|AKUA01000006.1|](http://www.ncbi.nlm.nih.gov/nucleotide/392094004?report=genbank&log$=nuclalign&blast_rank=30&RID=7G6ZGW3E014) | [gb|AKUA01000005.1|](http://www.ncbi.nlm.nih.gov/nucleotide/392100489?report=genbank&log$=nuclalign&blast_rank=32&RID=7G976GMH014) |
| ***M.abscessus_5S-0422_5S_0422*** | [gb|AKTZ01000012.1|](http://www.ncbi.nlm.nih.gov/nucleotide/392077834?report=genbank&log$=nuclalign&blast_rank=42&RID=7G22V81A01N) | [gb|AKTZ01000005.1|](http://www.ncbi.nlm.nih.gov/nucleotide/392092463?report=genbank&log$=nuclalign&blast_rank=44&RID=7G3H3JTH016) | [gb|AKTZ01000011.1|](http://www.ncbi.nlm.nih.gov/nucleotide/392087210?report=genbank&log$=nuclalign&blast_rank=43&RID=7G4PE5P701N) | [gb|AKTZ01000007.1|](http://www.ncbi.nlm.nih.gov/nucleotide/392090466?report=genbank&log$=nuclalign&blast_rank=42&RID=7G5VA7M901N) | [gb|AKTZ01000012.1|](http://www.ncbi.nlm.nih.gov/nucleotide/392077834?report=genbank&log$=nuclalign&blast_rank=33&RID=7G6ZGW3E014) | [gb|AKTZ01000004.1|](http://www.ncbi.nlm.nih.gov/nucleotide/392093063?report=genbank&log$=nuclalign&blast_rank=33&RID=7G976GMH014) |
| ***M.abscessus_5S-0304_5S_0304*** | [gb|AKTX01000007.1|](http://www.ncbi.nlm.nih.gov/nucleotide/392082724?report=genbank&log$=nuclalign&blast_rank=40&RID=7G22V81A01N) | [gb|AKTX01000005.1|](http://www.ncbi.nlm.nih.gov/nucleotide/392088603?report=genbank&log$=nuclalign&blast_rank=46&RID=7G3H3JTH016) | [gb|AKTX01000007.1|](http://www.ncbi.nlm.nih.gov/nucleotide/392082724?report=genbank&log$=nuclalign&blast_rank=44&RID=7G4PE5P701N) | [gb|AKTX01000005.1|](http://www.ncbi.nlm.nih.gov/nucleotide/392088603?report=genbank&log$=nuclalign&blast_rank=44&RID=7G5VA7M901N) | [gb|AKTX01000007.1|](http://www.ncbi.nlm.nih.gov/nucleotide/392082724?report=genbank&log$=nuclalign&blast_rank=31&RID=7G6ZGW3E014) | [gb|AKTX01000001.1|](http://www.ncbi.nlm.nih.gov/nucleotide/392091818?report=genbank&log$=nuclalign&blast_rank=34&RID=7G976GMH014) |
| ***M.abscessus_5S-0421_5S_0421*** | [gb|AKTY01000006.1|](http://www.ncbi.nlm.nih.gov/nucleotide/392079650?report=genbank&log$=nuclalign&blast_rank=41&RID=7G22V81A01N) | [gb|AKTY01000003.1|](http://www.ncbi.nlm.nih.gov/nucleotide/392089733?report=genbank&log$=nuclalign&blast_rank=45&RID=7G3H3JTH016) | [gb|AKTY01000006.1|](http://www.ncbi.nlm.nih.gov/nucleotide/392079650?report=genbank&log$=nuclalign&blast_rank=45&RID=7G4PE5P701N) | [gb|AKTY01000003.1|](http://www.ncbi.nlm.nih.gov/nucleotide/392089733?report=genbank&log$=nuclalign&blast_rank=43&RID=7G5VA7M901N) | [gb|AKTY01000006.1|](http://www.ncbi.nlm.nih.gov/nucleotide/392079650?report=genbank&log$=nuclalign&blast_rank=32&RID=7G6ZGW3E014) | [gb|AKTY01000002.1|](http://www.ncbi.nlm.nih.gov/nucleotide/392090669?report=genbank&log$=nuclalign&blast_rank=35&RID=7G976GMH014) |
| ***M.abscessus_M156*** | [gb|AKVU01000005.1|](http://www.ncbi.nlm.nih.gov/nucleotide/396586869?report=genbank&log$=nuclalign&blast_rank=49&RID=7G22V81A01N) | [gb|AKVU01000021.1|](http://www.ncbi.nlm.nih.gov/nucleotide/396586853?report=genbank&log$=nuclalign&blast_rank=27&RID=7G3H3JTH016) | [gb|AKVU01000011.1|](http://www.ncbi.nlm.nih.gov/nucleotide/396586863?report=genbank&log$=nuclalign&blast_rank=24&RID=7G4PE5P701N) | [b|AKVU01000014.1|](http://www.ncbi.nlm.nih.gov/nucleotide/396586860?report=genbank&log$=nuclalign&blast_rank=28&RID=7G5VA7M901N) | [gb|AKVU01000005.1|](http://www.ncbi.nlm.nih.gov/nucleotide/396586869?report=genbank&log$=nuclalign&blast_rank=49&RID=7G6ZGW3E014) | [gb|AKVU01000035.1|](http://www.ncbi.nlm.nih.gov/nucleotide/396586839?report=genbank&log$=nuclalign&blast_rank=38&RID=7G976GMH014) |
| ***M.abscessus_M148*** | [gb|AKVV01000117.1|](http://www.ncbi.nlm.nih.gov/nucleotide/396587102?report=genbank&log$=nuclalign&blast_rank=48&RID=7G22V81A01N) | [gb|AKVV01000199.1|](http://www.ncbi.nlm.nih.gov/nucleotide/396587020?report=genbank&log$=nuclalign&blast_rank=53&RID=7G3H3JTH016) | [gb|AKVV01000254.1|](http://www.ncbi.nlm.nih.gov/nucleotide/396586965?report=genbank&log$=nuclalign&blast_rank=23&RID=7G4PE5P701N) | [gb|AKVV01000043.1|](http://www.ncbi.nlm.nih.gov/nucleotide/396587176?report=genbank&log$=nuclalign&blast_rank=27&RID=7G5VA7M901N) | [gb|AKVV01000046.1|](http://www.ncbi.nlm.nih.gov/nucleotide/396587173?report=genbank&log$=nuclalign&blast_rank=48&RID=7G6ZGW3E014) | [gb|AKVV01000083.1|](http://www.ncbi.nlm.nih.gov/nucleotide/396587136?report=genbank&log$=nuclalign&blast_rank=37&RID=7G976GMH014) |
| ***M.abscessus_M139*** | [gb|AKVR01000001.1|](http://www.ncbi.nlm.nih.gov/nucleotide/396586757?report=genbank&log$=nuclalign&blast_rank=23&RID=7G22V81A01N) | [gb|AKVR01000002.1|](http://www.ncbi.nlm.nih.gov/nucleotide/396586756?report=genbank&log$=nuclalign&blast_rank=14&RID=7G3H3JTH016) | [b|AKVR01000007.1|](http://www.ncbi.nlm.nih.gov/nucleotide/396586751?report=genbank&log$=nuclalign&blast_rank=49&RID=7G4PE5P701N) | [gb|AKVR01000005.1|](http://www.ncbi.nlm.nih.gov/nucleotide/396586753?report=genbank&log$=nuclalign&blast_rank=50&RID=7G5VA7M901N) | [gb|AKVR01000013.1|](http://www.ncbi.nlm.nih.gov/nucleotide/396586745?report=genbank&log$=nuclalign&blast_rank=23&RID=7G6ZGW3E014) | [gb|AKVR01000006.1|](http://www.ncbi.nlm.nih.gov/nucleotide/396586752?report=genbank&log$=nuclalign&blast_rank=24&RID=7G976GMH014) |
